# Future perspectives in melanoma research: meeting report from the “Melanoma Bridge”, Napoli, December 5th-8th 2013

**DOI:** 10.1186/s12967-014-0277-z

**Published:** 2014-10-28

**Authors:** Paolo A Ascierto, Antonio M Grimaldi, Ana Carrizosa Anderson, Carlo Bifulco, Alistair Cochran, Claus Garbe, Alexander M Eggermont, Mark Faries, Soldano Ferrone, Jeffrey E Gershenwald, Thomas F Gajewski, Ruth Halaban, F Stephen Hodi, Richard Kefford, John M Kirkwood, James Larkin, Sancy Leachman, Michele Maio, Richard Marais, Giuseppe Masucci, Ignacio Melero, Giuseppe Palmieri, Igor Puzanov, Antoni Ribas, Yvonne Saenger, Bastian Schilling, Barbara Seliger, David Stroncek, Ryan Sullivan, Alessandro Testori, Ena Wang, Gennaro Ciliberto, Nicola Mozzillo, Francesco M Marincola, Magdalena Thurin

**Affiliations:** Istituto Nazionale Tumori, Fondazione “G. Pascale”, Napoli, Italy; Center for Neurologic Diseases, Harvard Medical School, Boston, MA USA; Translational Molecular Pathology, Earle A. Chiles Research Institute, Providence Cancer Center, Portland, OR USA; Departments of Pathology and Laboratory Medicine and Surgery, David Geffen School of Medicine at University of California Los Angeles (UCLA), John Wayne Cancer Institute, Santa Monica, CA USA; Center for Dermato Oncology, Department of Dermatology, University of Tübingen, Tübingen, Germany; Cancer Institute Gustave Roussy, Villejuif/Paris-Sud, Villejuif, France; Donald L. Morton Melanoma Research Program, John Wayne Cancer Institute, Santa Monica, CA USA; Department of Surgery, Massachusetts General Hospital, Harvard Medical School, Boston, MA USA; Department of Surgical Oncology, The University of Texas MD Anderson Cancer Center, Houston, TX USA; Departments of Medicine and of Pathology, Immunology and Cancer Program, The University of Chicago Medicine, Chicago, IL USA; Department of Dermatology, Yale University School of Medicine, New Haven, CT USA; Department of Medical Oncology, Dana-Farber Cancer Institute, Boston, MA USA; Westmead Institute for Cancer Research, Westmead Millennium Institute and Melanoma Institute Australia, University of Sydney, Sydney, NSW Australia; Division of Hematology/Oncology, Departments of Medicine, Dermatology, and Translational Science, University of Pittsburgh School of Medicine and Melanoma Program of the Pittsburgh Cancer Institute, Pittsburgh, PA USA; Royal Marsden NHS Foundation Trust, London, UK; Department of Dermatology, Oregon Health Sciences University, Portland, OR USA; Medical Oncology and Immunotherapy, Department of Oncology, University Hospital of Siena, Istituto Toscano Tumori, Siena, Italy; Molecular Oncology Group, The Paterson Institute for Cancer Research, Wilmslow Road, Manchester, M20 4BX UK; Department of Oncology-Pathology, The Karolinska Hospital, Stockholm, Sweden; Centro de Investigación Médica Aplicada, Clinica Universidad de Navarra, Pamplona, Navarra Spain; Unit of Cancer Genetics, Institute of Biomolecular Chemistry, National Research Council, Sassari, Italy; Vanderbilt University Medical Center, Nashville, TN USA; Tumor Immunology Program, Jonsson Comprehensive Cancer Center (JCCC), David Geffen School of Medicine, University of California Los Angeles (UCLA), Los Angeles, CA USA; Division of Hematology and Oncology, Tisch Cancer Institute, Department of Dermatology, Icahn School of Medicine at Mount Sinai, New York, NY USA; Department of Dermatology, University Hospital, West German Cancer Center, University Duisburg-Essen, Essen, Germany; Martin Luther University Halle-Wittenberg, Institute of Medical Immunology, Halle, Germany; Cell Processing Section, Department of Transfusion Medicine, Clinical Center, NIH, Bethesda, MD USA; Center for Melanoma, Massachusetts General Hospital Cancer Center, Harvard Medical School, Boston, MA USA; Istituto Europeo di Oncologia, Milan, Italy; Division Chief of Translational Medicine, Sidra Medical and Research Centre, Doha, Qatar; Sidra Medical and Research Centre, Doha, Qatar; Cancer Diagnosis Program, National Cancer Institute, NIH, Bethesda, MD USA; German Cancer Consortium (DKTK), Heidelberg, Germany

## Abstract

The fourth “Melanoma Bridge Meeting” took place in Naples, December 5 to 8^th^, 2013. The four topics discussed at this meeting were: Diagnosis and New Procedures, Molecular Advances and Combination Therapies, News in Immunotherapy, and Tumor Microenvironment and Biomarkers.

Until recently systemic therapy for metastatic melanoma patients was ineffective, but recent research in tumor biology and immunology has led to the development of new targeted and immunotherapeutic agents that prolong progression-free survival (PFS) and overall survival (OS). New therapies, such as mitogen-activated protein kinase (MAPK) pathway inhibitors, like BRAF and MEK inhibitors, as well as other signaling pathways inhibitors, are being tested in metastatic melanoma either as monotherapy or in combination, and have yielded promising results.

Improved survival rates have also been observed with immune therapy for patients with metastatic melanoma. Immune-modulating antibodies came to the forefront with anti-CTLA-4, programmed cell death-1 (PD-1) and PD-1 ligand 1 (PD-L1) pathway blocking antibodies that result in durable responses in a subset of melanoma patients. Agents targeting other immune inhibitory (e.g., Tim-3) or immune stimulating (e.g., CD137) receptors and other approaches such as adoptive cell transfer demonstrate clinical benefit in melanoma as well.

This meeting’s specific focus was on advances in targeted therapy and immunotherapy. Both combination targeted therapy approaches and different immunotherapies were discussed. Similarly to the previous meetings, the importance of biomarkers for clinical application as markers for diagnosis, prognosis and prediction of treatment response was an integral part of the meeting. Significant consideration was given to issues surrounding the development of novel therapeutic targets as further study of patterns of resistance to both immunologic and targeted drugs are paramount to future drug development to guide existing and future therapies. The overall emphasis on biomarkers supports novel concepts toward integrating biomarkers into contemporary clinical management of patients with melanoma across the entire spectrum of disease stage. Translation of the knowledge gained from the biology of tumor microenvironment across different tumors represents a bridge to impact on prognosis and response to therapy in melanoma.

## Introduction

**Figure 1:**
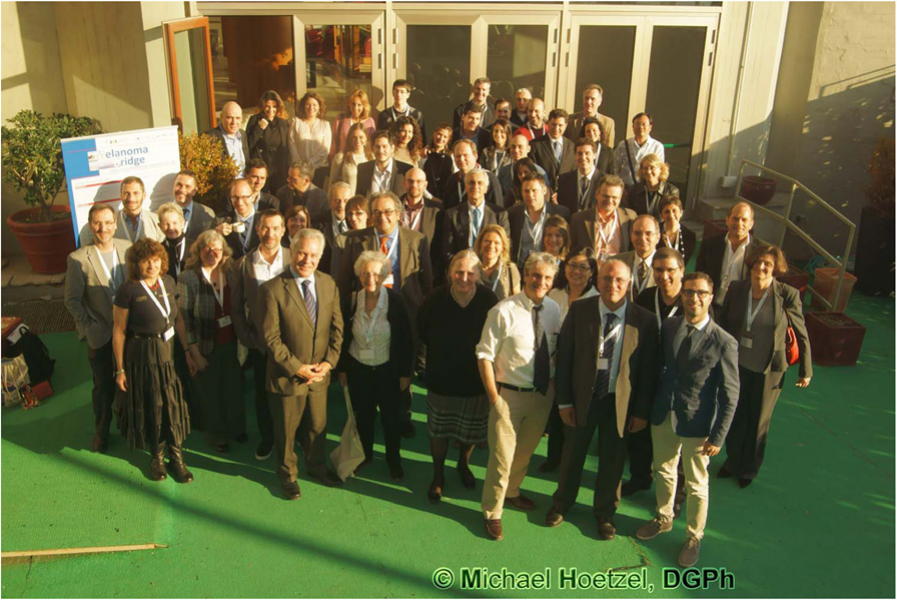
**Faculty and attendees of the 2013 Melanoma Bridge Meeting in Naples.**

The Melanoma Bridge 2013 meeting began on December 5^th^, 2013 by acknowledging the recent passing of Professor Natale Cascinelli by the organizers and all participants (Figure 1). Professor Cascinelli was one of the best known experts in melanoma in Europe. He was a scientific director of the National Institute of Oncology in Milan and was an active member of the Italian Ministry of Health, World Health Organization and Alliance against cancer among others.

Until 2011, dacarbazine (DTIC), interleukin (IL)-2 and interferon (IFN)α-2b were the only Food and Drug Administration (FDA) approved agents for the treatment of metastatic melanoma. Other single chemotherapy agents or angiogenesis inhibitors and combinations demonstrated modest activities. However, a true breakthrough in treatment of melanoma patients was the publication of the results from the phase 3 randomized trials of ipilimumab [1] and vemurafenib [2]. These trials demonstrated for the first time the benefit for melanoma patients as the treatment significantly improved overall survival (OS) and progression free survival (PFS) as compared with patients receiving chemotherapy in the control arms. Both vemurafenib and ipilimumab were FDA approved in 2011 and were added to dacarbazine and fotemustine (in Europe), as standard therapies available for metastatic melanoma patients.

The mitogen-activated protein kinase (MAPK) cascade is a critical intracellular signaling pathway that regulates cellular functions including proliferation, cell cycle regulation, survival, angiogenesis, and cell migration. The fundamental role of the RAS/RAF/MEK/ERK MAPK pathway in these cellular functions underlies its importance in oncogenesis and growth of melanoma cells [3]. Activating mutations in serine–threonine protein kinase BRAF, a constituent of the MAP kinase signal transduction pathway, have been identified in about 50% of patients with advanced melanoma [4]. The most commonly observed BRAF V600E mutation accounts for 90% of the mutations found in all patients with cutaneous melanoma, while other mutations (e.g., V600K, V600D, etc.) account for the remaining 10%. Mutated BRAF phosphorylates and activates MEK proteins (MEK1 and MEK2) which then activate downstream MAP kinases cascade. Mutated BRAF is the target of vemurafenib, a small molecule that inhibits the signal transmission between BRAF and MEK within the MAP-kinases pathway. This drug results in dramatic responses with a rapid improvement of symptoms and performance status following a decrease in metabolic rate and reduction of tumor size. The BRIM-3 trial, a phase 3 trial of vemurafenib vs. dacarbazine as first-line therapy for BRAF-mutated metastatic melanoma, demonstrated the advantage in OS of the targeted therapy vs. the chemotherapy with respect to overall survival (13.2 vs. 9.2 months). Most frequent toxicities of vemurafenib that patients experience include arthralgia, cutaneous rash, photosensitivity reaction, and squamous cell carcinoma/keratoacanthoma that are observed in 26% of patients [2].

Several new agents targeting BRAF and other downstream molecules in the RAS/RAF/MEK/ERK pathway are being tested in the clinic as single agents and in combination. Dabrafenib (Tafinlar) was FDA approved in 2013 for treatment of melanoma patients with BRAF V600E or V600K mutations. Dabrafenib seems also to be quite effective in the treatment of intracranial metastases [5]. Selective MEK inhibitors inhibited growth and induced cell death in BRAF mutated melanoma cell lines [6]. Trametininb (Mekinist), a potent MEK1/2 inhibitor received FDA approval in 2013 for treatment of patients with metastatic melanoma harboring BRAF V600E/K mutations. In Phase 3 clinical trial METRIC, patients were assigned to receive either trametinib or chemotherapy (decarbazine or paclitaxel). Patients treated with trametinib demonstrated significant improvement in PFS and OS. Currently, trametinib is not indicated for patients who have received previous BRAF inhibitor therapy.

NRAS mutations are observed in 15–25% of melanomas and are mutually exclusive with BRAF mutations; the latter of which are reported in 40–60% of patients with cutaneous melanoma [7,8]. BRAF and NRAS mutations both activate downstream MEK kinase that serves as the gatekeeper of extracellular signal-regulated kinase (ERK) activation. MEK inhibitor (MEKi) confers a preferential sensitivity to MEK kinase and thus it is a highly attractive target for melanoma treatment, as majority of tumors have mutations in either RAS (K-, N- or H-) or BRAF.

The combination of the BRAF inhibitor dabrafenib and the MEK inhibitor trametinib has been designed to delay the development of resistance to treatment with BRAF inhibitor (BRAFi), and to minimize toxic effects associated with BRAF inhibition. In 2013, the results of the phase 2 randomized trial of the combination of dabrafenib and trametinib have been published [9]. The data showed an important advantage of the combination in PFS vs. dabrafenib alone with 9.4 vs. 5.8 months, respectively. It received accelerated FDA approval in 2014 for use in the treatment of patients with metastatic melanoma with BRAF V600E and V600K mutations. Phase 3 randomized controlled trials comparing dabrafenib plus trametinib with dabrafenib or vemurafenib are ongoing. Depending on the results from these trials, the combination treatment might prove to be more favorable as compared to monotherapy with BRAF and MEK targeting agents. Studies comparing combinations of other BRAF and MEK inhibitors in phase 3 trials are also ongoing. At the 2013 ESMO/ECCO congress the data regarding the combination of vemurafenib and cobimetinib (MEK inhibitor) were presented [10]. These data demonstrated 85% objective responses (10% complete responses, 75% partial responses in metastatic BRAF-mutated melanoma), 13% stable disease and 2% progressive disease, while at a median follow up of 10 months median PFS was still not reached. A third combination of a BRAF inhibitor and a MEK inhibitor, LGX818 plus MEK162 was presented at the 2013 ASCO Congress with very encouraging preliminary data in BRAF-naïve and pretreated patients [11].

In the recent years, the blockade of immune checkpoints gained attention as one of the most promising approaches to activate anti-tumor immunity. Antigen presentation to T cells involves contact between T cell receptor (TCR) on T lymphocytes (TCL) and MHC of the Antigen Presenting Cells (APC). In addition, to activate the immune response binding between ligands B7-1 or B7-2 on APC and the co-stimulatory receptor CD-28 on TCL is required. To avoid excessive or auto immune T cell responses receptors that inhibit the immune response including cytotoxic T-lymphocyte associated antigen-4 (CTLA-4), are expressed on the T cell surface. Thus, targeting inhibitory immune receptors on activated T cells seems to be a logical approach to activate host immune response. Monoclonal antibodies (MAbs) targeting T cell checkpoint receptors such as CTLA-4, programmed cell death-1 (PD-1) receptor and others have shown great promise against advanced melanoma as well as lung and kidney cancers. Ipilimumab and tremelimumab are fully humanized MAbs targeting CTLA-4 receptor. Blocking of CTLA-4 results in enhancement of immune responses that are dependent on CD8^+^ effector T cells and CD4^+^ T cells, and inhibition of the suppressive function of T regulatory cells (Tregs). Phase 3 clinical trial comparing combination of ipilimumab plus gp100 glycoprotein vaccine vs. ipilimumab and the vaccine alone resulted in 1-year survival of 43.6%, 45.6%, and 25.3% patients, respectively. Two-year survival rates were 21.6% among patients who received combination therapy, 23.5% who received ipilimumab alone, and 13.7% among those who received gp100 alone [1]. Toxicity of ipilimumab is immune-mediated and most frequent side effects result diarrhea, pruritus, and, less frequently endocrinopathies and autoimmune hepatitis.

PD-1 is another inhibitory receptor on activated and exhausted T cells. Its two ligands PD-L1 and PD-L2 have distinct expression profiles. PD-L1 is expressed on a variety of immune and non-immune cells including various tumor types while PD-L2 is mainly expressed on antigen presenting cells. Blocking of the PD-1/PD-L1 interaction by administration of anti-PD-1 or anti-PD-L1 MAbs can activate T cells and enhance adaptive anti-tumor immune responses. Nivolumab [12] and MK-3475 (pembrolizumab formerly lambrolizumab) [13] are humanized MAb that block the interaction between PD-1 and its ligands and demonstrate durable responses in patients with advanced melanoma. As monotherapy MK3475 and nivolumab resulted in 37% and 31% objective response rate (ORR), respectively. In nivolumab-treated patients, 1 and 2 year survival was observed in 62% and 43% patients, respectively. Furthermore, the phase 1 trial of the combination of ipilimumab and nivolumab [14] demonstrated 40% ORR that can be compared to the ORR with vemurafenib (53%) and an estimated 1 year survival rate 82% in BRAF mutated patients. A phase 3 trial comparing concurrent nivolumab plus ipilimumab vs. either agent alone has been activated and is recruiting patients (NCT01844505). Pembrolizumab (Keytruda) received recently (2014) FDA approval for patients with unresectable or metastatic melanoma and nivolumab is expected to be approved by the FDA as soon as recent trial results are mature. In addition, treatment with anti-PD-L1 antibodies (e.g., MPDL 3280A and BMS-936559) was associated with durable responses with manageable toxic effects [15,16]. Overall, PD-1/PD-L1 blocking agents have shown an overall higher RR than ipilimumab and represent highly promising therapeutic options for melanoma patients.

Adoptive Cell Transfer therapy (ACT) yielded promising results in patients with variety of cancers including melanoma. There are many forms of ACT therapies being developed and sufficient amount of data demonstrate that expanded tumor infiltrating cells (TILs) or genetically engineered lymphocytes recognizing specific tumor can have potent, long-lasting effect-and can even eradicate some tumors entirely. One approach to immunotherapy involves patients’ own immune cells that recognize and attack their tumors. Although this approach has been restricted to small clinical trials so far, treatments using TILs have generated remarkable responses in patients with advanced cancer [17]. Lymphocytes can also be genetically engineered using viral vectors to produce MAb based receptors on their surface called chimeric antigen receptors (CARs). CARs are proteins that allow the T cells to recognize a specific antigen on tumor cells. Other approaches focus on TCR modified ACT therapy that involves introducing new TCR receptors that allow them to recognize specific cancer antigens. Both CAR and TCR therapies are being tested in patients with a variety of cancers and move closer to the mainstream; the next step will be investigating whether and how to integrate them with other cancer immunotherapies [18].

In summary, in just over 2 years, several agents have demonstrated an overall survival advantage for the treatment of patients with metastatic melanoma. Several drugs have been approved for the treatment of advanced melanoma and new class of agents has recently been shown to lead to durable responses in a substantial number of patients. There are now five categories of FDA-approved agents for these patients, including chemotherapy (dacarbazine), cytokines (IL-2 and IFNα), targeted therapy drugs including BRAF inhibitors (dabrafenib, vemurafenib) and MEK inhibitor (trametinib), immunotherapy agents anti-CTLA-4 (ipilimumab) and anti-PD-1 (pembrolizumab) MAbs, and the combination (dabrafenib and trametinib). In the coming years, it is reasonable to expect that another MEK inhibitor (MEK162), a third BRAF inhibitor (LGX818), another anti-PD-1 antibody (nivolumab) and an anti-PD-L1 antibody (MPDL3280A) will join the ranks of approved agents in melanoma.

Critical areas requiring rapid progress include efficiently combining the “best in class” drugs that already have demonstrated single-agent activity rather than multiplication of trials using drugs of the same classes. For example, vemurafenib and ipilimumab are drugs with different mechanisms of action and different kinetics of response: vemurafenib demonstrates quick action with rapid metabolic shutdown but with a response lasting only 6–8 months while ipilimumab acts slowly but is able to stabilize the disease. Thus, there is a strong rationale to consider immunotherapy in combination with molecularly targeted therapy for melanoma. Combinations that include immune checkpoint inhibitors and targeted therapy drugs are testing this hypothesis in clinic. While more work is required to achieve the goal of treating patients with regimens that are associated with durable remissions in the great majority of patients, there is hope that this goal will be achieved in the relatively near future.

### Diagnosis and new procedures

Established biomarkers for melanoma include the morphological and histopathological characteristics of the primary tumor. However, prognosis and risk for recurrence at any stage is only partially explained by parameters such as primary tumor localization, patient gender and age, mitotic rate, tumor thickness and ulceration. The pathological diagnosis of melanoma can be difficult as no single histological attribute can reliably be utilized to distinguish between nevus, borderline lesions and melanoma. In addition, the clinical heterogeneity of melanoma requires the development of classification schemes that use different factors to capture the various subtypes of melanoma. This ongoing process of tissue as well as serum biomarker identification and validation is resulting in a rapidly changing molecular view of cutaneous melanoma, which holds the promise of improving diagnostic and prognostic classification systems. However, significant challenges still remain to optimize and validate the molecular diagnostics for melanoma.

Five to ten per cent of individuals with melanoma have a family history of melanoma. In families with melanoma, affected individuals often have the atypical mole syndrome phenotype. About a quarter of all families with a hereditary pattern of melanoma have been linked to mutations in the tumor suppressor gene CDKN2A/p16 on chromosome 9p21, which affects the germline DNA and is therefore transmitted through generations. The p16 mutation carriers have exceptional risk to develop a melanoma, and the environment is likely to modify risk in p16 mutation carriers. CDKN2A/p16 has different penetrance depending on the geographical environment, ranging from 58% in the United Kingdom to 76% in the USA, and 91-92% in Australia [19]. Unfortunately, compliance with prevention recommendations is poor in this high-risk population, despite education, though it has recently been shown that p16 mutation carriers increase compliance with photo protection, self-skin examinations, and screening examinations following receipt of genetic counseling and genetic test results [20–21]. A study reporting the effects of melanoma genetic counseling and test reporting on screening adherence among unaffected carriers two years later has been recently published [22]. The population of this trial was composed by two large p16 mutated families, divided in 3 study groups (Unaffected non-carriers, Unaffected carriers and Affected carriers) with adherence reports obtained at baseline prior to reporting, immediately following test reporting, and at 1 month, 6 month, 1 year and 2 year time points to evaluate the long-range impact on prevention and screening. Unaffected carriers had durable improvement in the use protective clothing at 2 years and similar improvements were observed in daily routine use of photo protection and reduction of sunburns. In this population group even an improvement in Total Body Skin Examinations (TBSEs) was observed, especially compared with affected carriers. Similar improvements were reported for the number of Self Skin Examination (SSEs) and sites of SSEs at 2 years. So this study concluded that genetic test reporting has a significant, durable effect on compliance with some, but not all prevention recommendations and unaffected p16 carriers have the most to gain and demonstrate the most dramatic effect. It is not clear why provision of a personalized genetic result might empower behavioral change more than counseling alone.

In patients with excised cutaneous melanoma (CMM) appropriate follow-up testing has a higher impact on survival than adjuvant treatment. Lymph node (LN) ultrasound and protein S100ß levels in the peripheral blood are the key examinations. PET-CT demonstrated to be superior to conventional CT in detecting new metastatic lesions. The percent of patients with recurrences correlates with clinical stage, ranging from 0.4% for patients with stage IA to 30.3% for patients with stage IIIB melanoma. Recurrences can be considered “early” when the lesions are no more than 2 cm in diameter and there are less than 10 nodules that are totally surgically removable. “Late” recurrences are all other kind of metastases including LN, in transit, visceral etc. Overall survival of patients with recurrences vary depending on the early or late recurrence, and is more favorable for the first group. Survival benefit partially depends on the earlier time point of diagnosis and a resulting longer observation period between diagnosis and subsequent relapse or death (29.4% vs. 15.9% patients alive after recurrence at a median follow-up of more than 70 months in early and late recurrence groups, respectively). In a prospective study in 1288 melanoma patients, palpation of subcutaneous lymph nodes gave false-negative results in 68 of the 238 cases of histopathologically proven metastases (28.6%) [23]. Clinical examination was the least sensitive in the supraclavicular, axillary, and infraclavicular regions. The sensitivity and specificity of ultrasound examination were 89.2% and 99.7%, respectively, and clinical examination was determined to be 71.4% sensitive and 99.7% specific. Lymph node ultrasound recognizes 1/3 of lymph nodal metastases earlier than palpation and is recommended in stages IB – IIIC. Thus, the staging of primary melanoma for stage IA (≤1 mm) patients should be performed by physical examination (palpation) and dermoscopy, while for stage IB – IIB should include physical examination (palpation), dermoscopy, lymph node ultrasound and detection of protein S100ß in peripheral blood. For stage IIC – IIIC melanoma patients staging should be performed by physical examination (palpation), dermoscopy, lymph node ultrasound, detection of protein S100ß in peripheral blood, brain MRI and PET-CT scan or whole body CT scan.

Many serum biomarkers have been evaluated in CMM and several proteins and gene expression levels can be considered as potential biomarkers [24]. However, they remain to be clinically validated in order to provide the rationale for diagnosis, prognosis and the follow-up. Serum levels of Lactate dehydrogenase (LDH), Alkaline phosphatase (AP), and Melanoma Inhibitory Activity (MIA) as well as Tyrosinase (Tyr) and MelanA/Mart1 detected by RT-PCR have been shown to correlate with clinical stage and tumor progression. Protein S100ß at the cut off value of 0.12 μg/dl has a diagnostic accuracy of 0.84 (0.29 sensitivity – 0.93 specificity), MIA at the cut off value of 10.49 ng/dl has a diagnostic accuracy of 0.86 (0.22 sensitivity – 0.97 specificity), LDH at the cut off value of 240 U/L has a diagnostic accuracy of 0.77 (0.02 sensitivity – 0.90 specificity), AP at the cut off value of 168 IU/L has a diagnostic accuracy of 0.79 (0.17 sensitivity – 0.89 specificity) and RT-PCR (at the cutoff for Positive/Negative) has a diagnostic accuracy of 0.72 (0.24 sensitivity – 0.80 specificity). S100ß has been considered prognostic marker in metastatic melanoma as it is more related to the tumor burden and thus reflects both clinical stage and tumor progression. Although it seems to be limited to advanced stage III and stage IV melanoma patients and does not provide independent prognostic information it has become the most useful melanoma marker in clinical practice. Combining the prognostic value of LDH and S100ß it is possible to create 4 groups of melanoma patients with different prognosis and risk for relapse.

Detection of first relapse in stage III melanoma changes depends on the site of metastasis. Systemic metastasis are discovered in 53% of patients with the imaging exams, while nodal and local/in transit relapses are detected by the patients or their families in 48.6 and 62.5%, respectively. Several recommendations for follow-up schedules have been issued in Germany since 1990s regarding the length of the follow-up for melanoma patients but it did not reach international consensus. A recommendation of 10-years risk adapted follow-up with reduction of frequency of visits in the course of observation was recommended by some authors, while a long-term or lifelong follow-up based on risk of development of secondary CM has been proposed by others. An intense follow-up should be performed in the first 3 years post diagnosis as 80% of the recurrences occur in this period. Regular follow-up can be completed at 10 years. In patients with individual risk factors (dysplastic nevus syndrome, history of familial melanoma) dermatologic examinations should be performed lifelong. For stage IA follow-up visits should be performed every 6 months for the first 3 years and yearly until the completion at 10 years. Stage IB – IIB patients should be examined every 3 months for the first 3 years, every 6 months for the next 2 years and yearly until the completion at 10 years. For stage IIC – IV melanoma patients, follow-up visits should be performed every 3 months for the first 6 years and every 6 months until the completion at 10 years [25].

Concluding, the early detection of recurrences and the diagnosis of secondary melanomas is the goal of follow-up examinations, because this has an impact on overall survival of the patients. Frequency and scale of surveillance examinations should be adapted to the stage of the disease. In patients with medium risk for recurrence lymph node ultrasound and protein S100ß levels are recommended while in stage III patients regular imaging examinations are more appropriate.

For classic melanocytic lesions histological diagnosis is very accurate (>97%). Real problems begin with borderline lesions as melanocytic tumors of unknown malignant potential (MELTUMPs), atypical nevoid and spitzoid lesions, atypical cellular blue nevi, deep penetrating nevi, pigmented epithelioid melanocytomas etc. Predicting the biology of borderline lesions is difficult and may be incorrect and confound therapy, so adjunctive techniques are required accurately to diagnose such lesions and allow a better understanding of their etiology and relationships. To better understand the differences between borderline lesions Gene Expression Micro-arrays (GEM) can be used. This technique is performed by micro-dissection of the sample to separate tumor from normal non-tumorous tissue. Total mRNA is isolated, amplified and labeled before hybridization to human gene chip arrays to seek differentially expressed genes (>/=x 2 difference of gene expression in subgroups at p < 0.05 level of significance). Differential gene expression profile for primary melanoma and melanoma metastatic to sentinel lymph nodes using DNA micro array technology has been studied [26]. Genes involved in melanoma progression and early (lymphatic) metastases have been analyzed to identify gene markers with potential for prediction of prognosis, therapeutics, and diagnostics. A significantly lower level of gene expression was observed in metastases. e.g., Sentinel LN (SLN) metastases had fewer “up-regulated” genes relative to primary cutaneous melanoma. “Up regulated” genes showed less significant fold-change than “down- regulated” genes. Decreased gene products included encoded protein products involved in cell adhesion, cellular structural integrity and tumor suppression (e.g., Gap junction proteins, keratins, and stratifin), while MAGE proteins showed increased expression in SLN metastases. A non-protein coding nuclear RNA product of XIST was also increased in the metastases. Information gained in this study may help to understand the molecular events that underpin lymphatic invasion and the development of nodal metastases and may lead to biomarkers to identify primary lesions with the potential for lymphatic extension. Increased understanding of the molecular mechanisms of melanoma progression will contribute to improvements in treatment, diagnosis, and prognostic prediction.

Another ongoing project showed differential gene expression in the sentinel node that was predictive of non-sentinel node tumor status [Huang et al. in preparation]. SNL biopsy is widely used in the management of patients with melanoma. Results of the MSLT2 trial are still pending, and all patients with SN metastases are currently offered immediate completion lymph node dissection (CLND). However, not all patients with SN metastases will gain benefit from immediate CLND. So the problem is to identify patients likely to benefit from early nodal surgery. GEM has been used to investigate metastatic melanoma in SN from 15 patients who had immediate CLND: 7 with no melanoma in non-sentinel nodes (NSN) and 8 with metastasis-positive NSN. Significantly different levels in expression of 1232 genes have been found comparing these two groups. Combination of gene signatures, with clinical-pathological features, can enhance accuracy of prediction of NSN tumor status, separating patients likely to benefit from CLND, from those unlikely to so benefit. This has the potential to spare many patients the morbidity of unnecessary additional surgery and significantly reduce costs and post-surgery side effects, with improvement in quality of life. In future, enhanced staging will identify patients at high risk of visceral metastases who may benefit from adjuvant therapy.

GEM has also been used to compare conventional melanoma (cMM), nevoid melanoma (NM) and benign atypical nevi (BAN) [Sarantopoulos et al. in preparation]. Nevoid melanoma (NM) is a melanoma (MM) variant that mimics atypical nevi (AN) clinically and microscopically. Characteristics of NM can be very subtle and NM carries a high potential for misdiagnosis, treatment delay and medical and legal consequences. Currently, no tests exist that help differentiate atypical or “borderline” nevoid melanocytic lesions from nevoid melanoma. This study attempts to distinguish NM from AN and MM using gene micro array technology. Results of this study are expected to lead to development of novel molecular tests to separate these morphologically similar, but clinically disparate lesions. Although some commonality exists between nevoid and conventional melanomas discrete clustering of genes in NM relative to AN and MM suggests a distinctive molecular pathobiology for NM. One case of AN fell into NM’s gene expression territory on principle component analysis (PCA). Close histological examination of this outlier case did not show evidence of malignancy or changes suggestive of nevoid melanoma. The question whether this lesion is a potential pre-cursor to NM will only be settled by further investigation and clinical follow-up.

A further project is studying GEM Signatures of Spitzoid Lesions [Hillman et al. in progress]. These studies seek to determine whether there are genetic differences between Spitz nevus, spitzoid melanoma and atypical spitzoid tumors. Other questions are whether these genetic differences underpin the differing biology of these lesions and whether a genetic signature may be developed to be used as an adjunct to histology in separating these lesions. It may be possible to separate at least some spitzoid melanomas and some lesions diagnosed microscopically as either Spitz nevus or atypical spitzoid lesion vs. spitzoid melanoma using GEM to supplement clinical and microscopic examination. Further studies with clinical follow-up are needed to correlate differences in gene expression with clinical behavior in spitzoid melanoma and SM and other spitzoid lesions.

The 7^th^ edition of the AJCC melanoma staging system presented no major recommended changes for TNM and stage grouping criteria [27]. Mitotic rate was identified as an independent prognostic factor (patients with T1 melanoma included in T1b if at least 1 mitosis/mm^2^) and immunohistochemical detection of nodal metastases is also considered acceptable without a lower limit to designate N + disease. Stage IV analysis included more than 9900 patients and the overall revisions were approved by the UICC. The importance of mitotic rate as a prognostic factor for survival in stages I/II melanoma patients emerged from the analyses of the 2008 AJCC collaborative melanoma database. Moreover, in the 6^th^ edition of the AJCC melanoma staging system, stratification between T1a and T1b was based on ulceration and Clark level, while in the 7^th^ edition, stratification of patients with T1 melanoma is based on ulceration and number of mitoses/mm^2^ (≥1 vs <1). Evidence from multivariate survival analyses of patients with AJCC stages I/II melanoma showed that mitotic rate was the 2^nd^ most powerful independent predictor of survival after tumor thickness [28]. In another study, age was shown to be an independent predictor of survival for stages I, II and III melanoma; mitotic rate emerged as an independent predictor of survival except in patients <20 yrs and >70 yrs [29,30].

AJCC recommendations for N-staging included micro staging of all primary melanomas and pathological nodal staging for TIb-TIIc melanoma (since 2002) to minimize prognostic heterogeneity within stages and to incorporate SLN assessment into the staging system. The stratification of T1 disease into T1a and T1b melanoma continues to evolve. The technique of lymphatic mapping and sentinel lymph node biopsy has revolutionized the staging of melanoma [31]. The paradigm of nodal involvement has changed from macroscopic lymph node involvement to microscopic involvement with the implementation of lymphatic mapping and sentinel lymph node biopsy into treatment paradigms for patients with early-stage melanoma who are at risk for regional lymph node metastasis. Important prognostic factors among patients with regional node disease (stage III) include tumor burden, number of involved regional nodes, and primary tumor ulceration [27–32]. In a multivariate analysis for stage III melanoma patients with microscopic regional node involvement, important predictors of survival included the number of positive lymph nodes, tumor thickness, primary tumor ulceration, age, site of primary tumor, sex, and mitotic rate, while in patients with macroscopic involvement only number of positive lymph nodes and age were significant [32].

Despite tremendous strides in melanoma staging (e.g., AJCC/UICC), limitations exist in traditional staging systems regarding the ability to integrate various characteristics (patient, tumor, etc.), the inability to use continuous variables, and that estimates of survival are based only on time of diagnosis. Moreover, TNM-based staging generally applies to large cohorts of patients (and is therefore not truly individualized). To improve melanoma staging and prognosis, new statistical models and contemporary analytic approaches can be leveraged that better inform the use of multiple characteristics and continuous variables (such as mitotic rate, tumor thickness, or SLN tumor burden) or that estimate conditional survival probability after treatment and at any time during follow-up. Overall, this approach may significantly enhance our ability to combine prognostic features to better estimate cancer-specific survival in individual patient settings, ultimately by also integrating clinicopathological factors with molecular, immunological and/or host profiles [33].

### Molecular Advances and combination therapies

A greater understanding of the molecular biology of melanoma has guided much of the translational research in this disease and has led to the development of novel targeted therapies. Specifically, identification of alterations in signal transduction pathways has led to the development of a number of inhibitors of these pathways. Studies are underway combining different targeted agents, as well as combinations of immune therapy and signal transduction inhibitors. However, there is a growing concern over drug resistance to molecular therapies including BRAF inhibitors. Thus, novel targets for the treatment of metastatic melanoma are urgently needed.

Next generation sequencing (NGS) techniques for both whole exome sequencing (WES) and whole genome sequencing (WGS) have been used to characterize the overall genomic landscape of melanomas. These studies identify mutation signatures associated with melanomas with the most common well characterized driver genes e.g., BRAF, NRAS, KIT, GNAQ, or GNA11. In addition, mutations that co-occur with the most common driver mutations could contribute to disease progression. For example, mutations in p53 and COL1A1 genes more likely associate with BRAF whereas mutated PPP6C, KALRN, PIK3R4, TRPM6, GUCY2C, and PRKAA2 were found to be more frequently associated with NRAS mutated melanomas. Other potential driver genes were found in so called pan-negative melanomas that do not carry most common mutations including ALK, STK31, DGKI, RAC1, EPHA4, ADAMTS18, EPHA7, ERBB4, TAF1L, NF1, SYK, and KDR [34]*.*

Melanoma has a high prevalence of somatic mutations with more than 700 described in literature. Mutated “driver” genes confer growth advantage and are required for maintenance of the cancer cells. In contrast, “passenger” mutations do not confer clonal growth advantage and therefore do not contribute to cancer development. However, they might be carried along in the clonal expansion and therefore are present in all cancer cells. A central goal of cancer genome analysis is the identification of cancer genes that, by definition, carry driver mutations (oncogenes and tumor suppressor genes). A key challenge will therefore be to distinguish driver from passenger mutations. Exome sequencing has been successfully used to characterize mutational landscape of melanoma. Recently discovered mutated genes have highlighted new pathways as potential markers and therapeutic targets including RAC1 (RAS related rho family, small GTP binding protein), Neurofibromin 1 (NF1), and protein phosphatase 6 catalytic subunit (PPP6C).

A recurrent somatic missense mutation in RAC1 at the codon 29 that results in substitution of a proline to a serine residue (RAC1P29S) was discovered in up to 9% of sun-exposed melanomas in the Yale cohort. This discovery makes RAC1P29S the third recurrent most commonly mutated proto-oncogene in melanoma after BRAF and NRAS [35,36]. RAC1P29S is present in both BRAF/NRAS mutated and wild type cutaneous melanomas. Furthermore, RAC1P29S mutation is predominant in males (95%) and is associated with a signature of UVB induced DNA damage CCT > TCT. Most patients in the Yale cohort reported excess sun exposure and most of the primary melanoma lesions were in sun exposed areas. This mutation is not detected in congenital, benign or blue nevi. It is not present in the germline DNA of 1,700 melanoma patients tested out of the sample of 5,090 individuals from USA and a worldwide. RAC1P29S is the first recurrent, cancer-associated, gain-of-function mutation belonging to the Rho-family GTPase. It is a “fast-cycling” mutant, with increased inherent GDP → GTP nucleotide exchange with no effect on GTPase activity [37]. RAC1P29S displays increased binding to target kinases PAK1 and MAP3K11 (MLK3), increases membrane ruffling, cell proliferation and migration of melanocytes. Since RAC1 promotes melanoma cell proliferation this pathway can become a new target for therapy, but preclinical data and clinical trials confirming its clinical relevance are required.

The NF1 tumor suppressor gene encodes a RAS GTPase-activating protein neurofibromin, which negatively regulates RAS by catalyzing the hydrolysis of RAS-GTP to RAS-GDP. Consequently, RAS and downstream effector pathways can be aberrantly activated in NF1*-*deficient tumors. Inactivating mutations are frequent in NF1 and are more common in sun-exposed melanoma in older patients and in melanomas lacking BRAF/NRAS mutations [35]. Importantly, loss of function NF1 gene mutations can result in the sustained activation of the MAPK pathway which may render cells resistant to RAF and MEK inhibitors [38]. Together, these studies provide new insights into the signaling that underlies melanoma initiation and progression and suggest novel therapeutic strategies for patients whose melanomas are NF1-deficient.

Mutations in the catalytic subunit of protein phosphatase 6 (PPP6C) have been identified in ~12% of cutaneous melanomas suggesting that it may act as a driver gene. All mutations in PPP6C are in the active domain and most are in tumors with BRAF/NRAS mutations. Protein phosphatase 6 is reported to regulate NF-κB, DNA-dependent protein kinase (DNA-PK), histone γ-H2AX, and Aurora-A kinase. Mutations in PPP6C reduce phosphatase activity, leading to activation of oncogenic kinase Aurora-A which is the major PP6 substrate, resulting in chromosome instability and DNA damage [39]. This suggests that inhibition of Aurora-A kinase activity may be useful in the treatment of melanoma. Specific kinase inhibitors targeting Aurora-A and B already demonstrated promising results in clinical trials.

The power of high-throughput NGS such as exome sequencing is being harnessed in addressing an increasingly diverse range of biological problems including tumor heterogeneity, drug resistance, neoantigens, new markers and therapy targets.

The importance of the information generated through this method is application of NGS for routine clinical sequencing for patient’s selection for specific treatments, identification of resistance mechanism and designing alternative therapies.

Since 2011 three selective BRAF inhibitors (BRAFi) (vemurafenib, dabrafenib and LGX818) have been developed as monotherapy treatment and clinically tested in combination with MEKi and other compounds in melanoma. As a single agent LGX818 in the phase 1 escalation trial obtained disease control rates of 88% for BRAFi naïve patients (65% overall response rate, ORR) and 43% for patients previously treated with a BRAFi (11% ORR). Median PFS was 217 days (about 7.1 months; 95% CI, 135- > 337 days) in BRAFi naïve patients and 58 days (about 1.9 months) in patients previously treated with a BRAFi. Median duration of response (DOR) was estimated to be 45.3 weeks (95% CI, 17.1-not reached) for BRAFi naïve patients and 16.1 weeks (95% CI, 13.9-19.1) for BRAFi pretreated patients. Toxicity profile was favorable, especially compared with the other BRAFi with very low incidence of fever, arthralgia, photosensitivity reactions whereas squamous cell carcinoma (SCC) \Plantar-Palmar Hyperkeratoses appeared to be the most frequent toxicity of LGX818 [40].

However, the clinical use of BRAF inhibitors for treatment of metastatic melanoma is limited by the development of drug resistance. There are various mechanisms of acquired (late) BRAFi resistance through MAPK pathway reactivation such as BRAF amplification and alternative splicing, RAS mutations, CDKN2A mutations, activating mutations in MEK1/2 and NF1 loss [41]. Overexpression of Ser/Thr kinase COT encoded by MAP3K8 gene has also been described in the context of BRAF inhibitor resistance. Almost all these mechanisms lead to reactivation of MAPK pathway and dual inhibition of both MEK and BRAF is thought to circumvent treatment resistance that commonly occurs with single-agent therapy. Recently, a potent MEK1/2 inhibitor trametininb was developed for treatment of patients with unresectable metastatic melanoma harboring BRAF V600E/K mutations [42]. Another MEK inhibitor, MEK 162 is active in both in BRAF mutated and NRAS mutated metastatic melanoma. In phase 2 clinical trial MEK162 treatment resulted in a PFS of 4.8 months with a favorable toxicity profile. The most frequent toxicities have been in papulopustolar cutaneous rash, creatine phosphokinase (CPK) elevation, and visual disturbances [43]. GDC-0973 (cobimetinib), another MEK inhibitor was tested in previously untreated BRAF V600 mutation-positive patients with unresectable locally advanced or metastatic melanoma. Other clinical trials with agents blocking oncogenic MAPK pathway signaling include studies with two ERK 1 and 2 inhibitors, MK-8353 (formerly SCH 900353, Merck) and BVD-523 (BioMed Valley Discoveries, Inc.).

In 2014 the FDA approved the combination of the BRAF inhibitor dabrafenib (Tafinlar) and the MEK inhibitor trametinib (Mekinist) for patients with unresectable or metastatic melanoma who harbor a BRAF V600E or V600K mutation [9]. In the phase 2 trial BRF113220 of dabrafenib plus trametinib vs. dabrafenib alone, the targeted therapy combination demonstrated a higher response rate (76% vs. 54%, respectively). Patients progressing on dabrafenib could cross to the combination arm (45 of 54 monotherapy treated subjects crossed-over). Median PFS was 9.4 months for the combination vs. 5.8 months for dabrafenib alone. Phase 3 randomized controlled trials comparing dabrafenib plus trametinib with dabrafenib (NCT01584648) or vemurafenib (NCT01597908) are ongoing.

Preclinical data in a model of BRAF mutant melanoma also supported the combination of LGX818 and MEK162. In the dose escalation phase the doses to be used in the expansion phase of the combination of LGX818 at 600 mg and MEK162 at 45 mg were identified, according to the dose limiting toxicity (DLTs). Both BRAFi naïve and BRAFi pre-treated BRAF-mutated metastatic melanoma patients were enrolled [10]. PK profile of the combination was similar to that of the two monotherapies on both day 1 and day 15, no drug-drug interaction (DDI) was observed and drug exposures were similar in combination compared with single-agent studies of MEK162 and LGX818. The overall response rate was 89% for BRAFi-naïve and 21% for BRAFi-pretreated patients with melanoma and 67% for patients with PTC. As of July 1, 2013, 11 of 30 patients remained under study, including 7 of 9 patients with BRAFi-naïve melanoma (Range duration of exposure was 3.9-12.6 months), 3 of 14 patients with BRAFi-pretreated melanoma (Range duration of exposure was 0.2-12.6 months) and 1 of 7 patients with mCRC or PTC. Toxicity profile was mild and the only G.3 adverse event (AE) resulted in transaminase elevation in 2 patients (6.7%). Compared with the other two combinations LGX818 plus MEK162 were much more tolerable for cutaneous, gastrointestinal and hepatic side effects. Ocular toxicity rate was slightly higher than that seen with the combinations of dabrafenib plus trametinib and vemurafenib plus cobimetinib.

Another combination of vemurafenib and the MEK inhibitor cobimetinib (GDC0973) was tested in the phase 2 BRIM-7 trial [9]. BRAF inhibitor naïve mutated metastatic melanoma patients obtained 85% response rate with a disease control rate of 98%. At a median follow-up time of 10 months median (m) PFS was not reached, while in patients who progressed with BRAFi the PFS resulted in 2.8 months. Phase 3 trial of cobimetinib in combination with vemurafenib compared with vemurafenib alone in melanoma that will evaluate the treatment efficacy in previously untreated BRAF V600 mutation-positive patients with unresectable locally advanced or metastatic melanoma is ongoing (NCT01689519). The combination of the BRAF inhibitor vemurafenib and the MEK inhibitor cobimetinib significantly improved progression-free survival (PFS) compared with vemurafenib alone for patients with untreated BRAFV600-mutated advanced melanoma, according to the results from this study. Ninety nine percent of the tumors that had a complete remission with the BRAFi plus MEKi combination therapy will never progress. Progression occurs in 50% of the cases with new lesions, in 44% with the progression of pre-existing lesions, and in 6% with both.

However, resistance is a concern even with the combination of BRAFi plus MEKi treatment. Although, the withdrawal of the drugs administration might result in a rapid progression of the disease the potential strategy to reduce side-effects and to prolong PFS could be an intermittent dosing regimen. Intermittent therapy was demonstrated to prevent the onset of the resistance to BRAFi in a murine xenograft model [44]. A recent report also demonstrated that melanoma patients who developed resistance to dabrafenib or the combination of dabrafenib plus trametinib benefited from re-challenge with BRAF inhibition following treatment interruption [45]. Because MAPK and PI3K-AKT pathways are the predominant signaling pathways in melanoma and MAPK-independent resistance to BRAF inhibitors can be mediated through enhancement of signaling through the PI3K-AKT pathway. Activation of the PI3K-AKT pathway can be achieved by activating mutations in the signaling molecules, deletion of the phosphatase and tensin homolog (PTEN) or overexpression or over-activation of upstream receptor tyrosine kinases (RTKs) including PDGFRβ, IGFR-1 or EGFR. There are three distinct genes encoding unique isoforms of AKT located at loci 14q32, 19q13, and 1q44, encoding AKT1, AKT2 and AKT3, respectively. The three isoforms of AKT may each have distinct biological relevance which points to the need to selectively target AKT isoforms in clinical drug development. Phosphorylation activates AKT, which then triggers a number of downstream signals. Particularly relevant in cancer is stimulation of tuberous sclerosis complex 2 (TSC2) protein, which associates with tuberous sclerosis 1 (TSC1) and is a key mediator of the mammalian target of rapamycin (mTOR) [46]. Increased reliance on PI3-AKT pathway as resistance mechanism to BRAF/MEK inhibition suggests that co-targeting the MAPK and the PI3K-AKT pathways would be reasonable approach to achieve synergistic anti-tumor activity. Combining dabrafenib with an AKTi appears to be a promising strategy for more effective treatment of melanoma patients as non-MAPK acquired resistance may benefit from the addition of an AKTi. AKT inhibitors showed synergistic effect with BRAF inhibitors and are able to reverse resistance to combination of BRAF plus MEK inhibitors. This is the basis of a phase 1/2 clinical trial (SWOG S1221, NCT 01902173), which has the goal of determining the safety of the combination of dabrafenib and the clinical grade AKTi GSK2141795 in patients with BRAF mutant cancer. BRAF V600 mutant melanoma patients progressing on prior BRAF inhibitor therapy were enrolled in this trial. AKT inhibitors (such as GSK2141795B) or mTOR inhibitor such as rapamycin combined with vemurafenib or AZD6244 reverses resistance in patient-derived vemurafenib-primary/-acquired resistant cell lines [47]. Combination of vemurafenib and rapamycin analogs such as everolimus and temsirolimus is also tested in BRAF mutation positive malignant melanoma (NCT01596140).

Tumor intrinsic pathways such as Heat Shock Proteins (HSP) can also be targeted. Hsp90s are among the most abundant proteins in the cell. They make up 1-2% of total cellular protein, which can increase to 4-6% under stress. These proteins are essential for proper folding, maintaining stability and avoiding degradation of a large number of proteins. Hsp90s consists of a homodimer with three domains, N-terminal domain with ATP-binding site, middle domain which bind to client proteins and C-terminal, responsible for dimerization and binding to different co-chaperones. XL888 is a novel, water soluble, orally available inhibitor of HSP90 from Exelixis, Inc. that is able to induce *in vitro* apoptosis and cell cycle arrest in vemurafenib resistant cell lines, leading to degradation of key proteins implicated in resistance [48].

Other compounds are targeting MDM2. MDM2 can interfere with p53-mediated apoptosis and tumor growth arrest. Inhibition of MDM2 can restore p53 activity in cancers leading to anti-tumor effects with apoptosis and growth inhibition. Two small molecule inhibitors of MDM2 JNJ-26854165 [49] and RO5045337 [50] are in clinical development. p53 mutational status is a predictive biomarkers of MDM2 inhibitor sensitivity because only melanomas containing wild type p53 were sensitive to the drug while p53 mutated melanoma did not respond. Preclinical data demonstrate the synergistic effect of MDM2i plus BRAFi and combination of BRAFi with MEKi. Moreover combination of MDM2 and BRAF/MEK inhibition suppressed growth of human colon cancer cell line RKO tumor xenograft. These results have provided an encouraging direction for p53-target therapeutic strategy utilizing inhibition of MDM2.

Blocking key components of both the tumor and its micro-environment offers a novel combination therapy approach for melanoma. Because BRAF targeted therapy is associated with fast but short-lasting responses the combination with immune therapy which provides durable responses has been considered. The first study to combine immunotherapy and targeted therapy was a combination of vemurafenib with CTLA-4 check point inhibitor ipilimumab. This study was closed early due to an unexpected number of hepatotoxic events that were probably related to autoimmune toxicity [51]. In addition, treatment with BRAF inhibitors is associated with an enhanced melanoma antigen expression, CD8^+^ T cells infiltration into tumors and enhanced T cell cytotoxicity. Thus a phase 1 clinical trial of dabrafenib plus ipilimumab in BRAF-mutated metastatic melanoma patients was initiated in 2013 (NCT01767454). A triple combination arm of dabrafenib plus trametinib plus ipilimumab is planned in this trial. Other trials of sequencing immunotherapy with combination of targeted therapy have been designed (vemurafenib/high dose IL-2, NCT01683188).

Another strategy to target tumor microenvironment is directed towards intratumoral immunosuppressive myeloid cells, including myeloid derived suppressor cells (MDSCs) and macrophages is being explored in preclinical study. PLX3397 is a selective inhibitor of colony stimulating factor-1 receptor (CSF1R), Kit, and FMS-like tyrosine kinase 3 (Flt3) that down modulates the key cell types involved in tumor progression including macrophages. Inhibiting intratumoral macrophages with PLX3397 improves the antitumor activity of tumor-specific T cells. Blocking myeloid cells recruitment with PLX3397 improved the efficacy of TCR engineered ACT [52]. Clinical trial testing PLX3397 has been planned for treatment of solid tumors. The combination of intratumoral macrophage depletion with PLX3397 also synergizes with BRAFi. A clinical trial of the combination of PLX3397 and vemurafenib in BRAF mutated melanoma is currently open to accrual (NCT01826448).

Targeted melanoma therapy with new drugs, including selective BRAF-inhibitors, holds great promise; however this therapy is limited because of the onset of resistance in most patients within a year. Defective apoptosis pathways may be a barrier to effective systemic treatment of melanoma. The interest in anti-apoptotic proteins has increased with the development of small-molecule inhibitors such as ABT-737, ABT-263 (navitoclax, AbbVie, Inc.), ABT-199 (AbbVie, Inc.), and GX15-070 (obatoclax, Cephalon, Inc.).

The group of anti-apoptotic proteins is comprised of Bcl-2, Bcl-xL, Bcl-w, Bfl-1, and Mcl-1 members possessing four BH domains (BH1, BH2, BH3 and BH4). In addition to the BH family, another major group of proteins called the inhibitors of apoptosis (IAPs) plays a role in regulating the intrinsic pathway of programmed cell death. Particularly well-characterized members of this family include XIAP (X-chromosome-linked IAP), cIAP1 (cellular IAP1), cIAP2 (cellular IAP2), Apollon, ML-IAP (melanoma IAP), survivin, IAP-like protein 2 (ILP2), and NAIP (neuronal apoptosis inhibitory protein) [53]. Members of pro-apoptotic proteins group have been subdivided into two groups: the Bax subgroup that includes Bax, Bak, and Bok proteins, having three BH domains (BH1, BH2, and BH3) and the BH3-only group that includes Bid, Bad, Bik, Bim, Noxa, Puma, and Hrk.

Several apoptosis related proteins have altered expression in melanoma. Expression of anti-apoptotic mediators such as Bcl-2, Bcl-xl, Bcl-w, Mcl-1, Bfl-1/A1 and Bcl-B may affect prognosis and/or response to treatment. For example, high levels of Bcl-2 can be a marker of good prognosis considering that Bcl-2 expression is reduced in progressive lesions. Other studies resulted in inconsistent results demonstrating that high levels of Bcl-xL and Mcl-1 correlate with tumor progression and they are associated with poor prognosis. High levels of survivin in sentinel lymph nodes also indicated poor prognosis as high Bcl-2 and Bcl-xL may confer poor response to chemotherapy. Other Bcl-2 family members Mcl-1 and Bcl-2A1 are differentially expressed in melanoma cell lines and have been shown to be increased in the melanoma progression.

Strong preclinical data from numerous groups have shown that targeting apoptosis improves the efficacy of chemotherapy and molecularly targeted therapy. Subsequently, the Bcl-2 antisense agent oblimersen tested in a randomized trial comparing the combination of oblimersen with DTIC vs. single-agent DTIC showed improved survival in the subgroup of metastatic melanoma patients with a normal LDH. In phase 2 trial oblimersen in combination with DTIC obtained a higher objective response rate of 13.5 vs. 7.5 months, CR 2.8 vs 0.8 and PR 10.6 vs 6.8 months, and resulted in better outcome in metastatic melanoma patients with normal LDH levels than in patients with high LDH levels, but these data were not confirmed in phase 3 trials [54]. A confirmatory phase 3 trial in patients with normal LDH did not show a survival advantage of the combination, and clinical development of oblimersen was discontinued [55]. Mimetics of the second mitochondria derived activator of caspase (SMAC) are a new class of targeted drugs being developed for treatment of solid tumors and hematological cancers. SMAC mimetic/survivin inhibitor YM155 tested in phase 1 and 2 trials resulted in encouraging safety data, but little clinical activity in metastatic melanoma [56,57]. ABT-737 mimetic of the BH3 domain of the pro-apoptotic protein Bad and selectively binds and inhibits the anti-apoptotic proteins Bcl-2, Bcl-xL, and Bcl-w. The limited efficacy of ABT-737 as a single-agent therapy indicated that anti-apoptotic proteins are not effectively targeted by this drug. Mcl-1 and Bcl-2-related protein A1 (Bfl-1/A1), play an important role in survival of tumor cells [58] and small-molecule inhibitors of Mcl-1 may be more effective against melanoma. Several compounds reported to be active against Mcl-1 such as obatoclax, prodigiosin, maritoclax have become available. The principal rationale for use of BH3-mimetics has been to sensitize cancer cells to other drugs by reducing their threshold for apoptosis. BH3-mimetics combination with proteasome inhibitor bortezomib, alkylating agents, immunotoxins, or cytotoxic drugs appears promising.

In patients with BRAF mutated melanoma BRAF/MEK inhibitors have variable effects on Bcl-2 family protein expression resulting in increased levels of mRNA for Bim, Bid, Bcl-xL and Bcl-w, while decreased level of mRNA for Mcl-1 or unchanged level of mRNA for Bcl-2. BRAF/MEK inhibitors have fewer effects on Bcl-2 family protein expression, and *in vitro* lead to increased Bim:Mcl-1 interaction. BH3-mimetics facilitate activation of intrinsic death pathway and navitoclax and obatoclax are most complementary to BRAFi [59]. The synergy of the combination of BRAFi and navitoclax in BRAF mutant cell lines *in vitro* has been clearly demonstrated. *In vivo*, the combination of BRAFi and navitoclax is associated with increased magnitude and duration of the response. Combination of the BRAFi PLX4720 and ABT-737 was cytotoxic for melanoma cells synergistically *in vitro*, depending on the induction level of Bim and down regulation of Mcl-1 [60]. Importantly, this combination also synergistically induced apoptosis in cell lines derived from BRAF V600E tumors obtained from patients before treatment with BRAFi. A more recent study confirmed the synergy of MAPK inhibitors and BH3-mimetics both for melanoma and colon cancer cells [61].

ClinicalTrials.gov lists currently 21 trials for navitoclax and 19 for obatoclax. The most interesting combination for melanoma patients harboring a BRAF V600E mutation would be the combination of a BH3-mimetic with a selective BRAF inhibitor. Indeed, a currently recruiting trial is studying the side effects and best dose of dabrafenib, trametinib, and navitoclax in patients with melanoma (NCT01989585/CTEP P9466).

Locoregional interventions can markedly improve overall survival among melanoma patients whose disease already disseminated and metastasized. For many years surgical resection has been the only therapeutic option to impact on patient’s survival but even in the era of more effective therapies, locoregional treatment still remains a vital consideration to improve survival and reports suggest the curative effect of surgery in certain patients. Metastastectomy has been associated with favorable survival for patients with metastases in the liver, lung, bowel or spleen/adrenal gland. Appropriate selection of patients for surgical intervention is critical, and Tumor Volume Doubling Time (TVDT) and Disease-Free Interval (DFI) are considered prognostic factors. A more rapid TVDT corresponds generally to a shorter survival [62]. Median overall survival (mOS) of melanoma patients is about twice longer (30 vs 18 months) if they are stratified for DFI with a cut off of 36 months; patients with a longer DFI have longer mOS [63]. Other prognostic factors in surgically resected stage IV melanoma are the ability to achieve complete surgical resection of all detectable disease, number of metastatic lesions (solitary vs. multiple), initial site of metastasis and the stage. Survival of patients with more than one lesion is shorter than those with one metastasis [64] and the site of recurrence has a significant impact on the outcome of the patient [65]. Multiple single institution studies indicate longer survival following resection of multiple distant metastases for melanoma. Longer survival rates were demonstrated in these studies than in the data provided by the Korn meta-analysis regarding the efficacy of multiple cooperative group phase 2 studies in advanced melanoma [66]. Thus, these data support the role of surgery in the treatment of advanced melanoma. Novel targeted therapy and immunotherapy drugs such as vemurafenib and ipilimumab, respectively, can be integrated with surgical procedures, with an overall impact on patients’ survival as demonstrated in patients treated at the National Cancer Institute of Naples using such a multidisciplinary approach.

Metastasis can be considered a focus of resistance. Improved survival in metastatic melanoma patients after surgery suggests the presence of endogenous anti-tumor responses [67]. Tumor resection may be associated with reduced tumor burden and decreased tumor-induced immunosuppression. Although new effective drugs such as ipilimumab and vemurafenib can have significant impact with rapid improvement of symptoms not all metastatic sites respond to these therapies. Thus, for patients with mixed or partial responses surgery may be required to remove resistant lesions. In those who develop solitary or small number of metastases in the liver, lung, bowel or spleen/adrenal gland resection can obtain a significant impact on survival of the patient. Metastases can also be considered as a vaccine because they are HLA-matched and represent a “personalized” source of tumor antigens. Tumor cells elicit an anti-tumor immune response of which tumor antigen specific T cells are the key regulators. However, despite the presence of tumor specific T cells the tumor may continue to proliferate and metastasize. Tumor resection may augment patient’s endogenous immune responses and change the balance in favor of anti-tumor immunity resulting in increased efficacy of locoregional intervention. In the late 19^th^ Century, William Bradford Coley, a surgeon of New York, observed that unresectable sarcomas regressed after superinfection with erysipelas [68]. Injecting mixed toxins of streptococcus bacteria causing erysipelas and bacillus prodigious resulted in regression of disease in more than one hundred and forty patients. Using the same concept, different experiments have been performed with the intralesional injection of interferon, interleukin 2, GD2 ganglioside specific MAb, oncolytic herpes virus encoding GM-CSF or BCG that obtained about 80% of responses [69].

Electrochemotherapy (ECT) combined with other treatments can also provide improved modality therapy for melanoma patients. This technique combines chemotherapy (bleomicin or cisplatin) with electroporation induced by needles inserted into the metastatic lesions that give an electric impulse (electroporator). Electroporation performed with some intratumoral short and very high voltage electric impulses facilitate the opening of transmembrane channels that allow delivery of non-permeant drug to the cell interior. As a result of transient permeabilization of the cell membrane even high molecular weight chemotherapy agents (bleomicin), which normally can’t get inside can diffuse inside the cell. With this procedure bleomicin concentration inside the cell increases about 10000 times than in standard therapy resulting in apoptosis of cancer cells. Indications of ECT application are the following: in-transit disease not amenable to surgery (multiple lesions that are not > 3 cm); early cutaneous relapses after previous surgical treatment; complete or partial responses after previous ECT treatment; palliative purpose (i.e., hemostatic or painful lesions) or as a neoadjuvant (extensive lesions or to reduce surgical approach). The analysis of the database of the National Cancer Institute of Naples (160 patients treated with ECT for 224 treatments) shows that different types of cancer can be treated with ECT including melanoma, squamous cell carcinoma, basal cell carcinoma, breast cancer and others. ECT can also be combined with the new anti-melanoma drugs, ipilimumab and/or vemurafenib. Another form of locoregional treatment for metastasis in case of liver metastases is liver perfusion as palliative treatment [70].

Although systemic treatment approaches for melanoma continue to evolve including emerging therapies (targeted and immunotherapies) local therapies including surgery or liver perfusion play an important role in the multidisciplinary management of melanoma. Thus in the future, advanced melanoma patients might benefit the most from a multimodality treatment including chemotherapy, targeted therapy, immunotherapy, radiotherapy, surgery and ECT options.

Problems in combining targeted drugs are dosing, targeting and tumor evolution. Combination targeted therapies are designed to overcome drug resistance. After all, combination therapy is effective for infectious diseases e.g., HIV, bacterial infections and tuberculosis. Combination cytotoxics also work well for haematological malignancies/germ cell tumours even if they are less efficacious for advanced solid tumours and are not curative. Often oncologists have combined drugs simply because they can but combining drugs at full single agent dose almost always increases side effects. This is acceptable if cure rate is increased or survival prolonged, but in melanoma historically combination cytotoxic chemotherapy/immunotherapy increased toxicity without survival benefit. The combination of BRAFi plus MEKi however has shown impressive results in clinical trials. For example, vemurafenib and cobimetinib in the BRIM 7 trial gave an 85% objective response rate and 98% control disease rate [10]. Dabrafenib and trametinib showed a higher efficacy compared with dabrafenib with a lower toxicity related to paradoxical MAPK pathway activation, as discussed earlier [9]. So the combination of BRAFi plus MEKi is possible at the full dose and with a better safety profile.

Most targeted drugs have a dose–response relationship for efficacy and toxicity and a narrow therapeutic index and approved doses are often close to maximum tolerated dose (MTD); dose reduction therefore may lead to reduced efficacy. Combination very often leads to dose reduction because of toxicity. In some cancers such as RCC this is well illustrated and the same is true for example in combining MEKi with PI3K or AKT inhibitors: full dosing is not possible. Dose reduction might not be a problem if you know that tumour A in patient B at time point C in metastasis D only needs 25% inhibition of pathway E (e.g. PI3K) and 50% inhibition of pathway F (e.g. MAPK). This is not easy information to gather currently. Relatively non-invasive tests are obviously needed to acquire this information and even ‘liquid biopsies’ still need to be validated with a gold standard.

Under the selective pressure of therapy tumours evolve to be resistant to treatment. So intratumoral heterogeneity is another problem to combine anti-cancer drugs; in advanced RCC, 65% mutations may be heterogeneous and not present in every biopsy [71]. Intratumoral heterogeneity/branched evolution can be considered a substrate for Darwinian selection and this has been demonstrated in multiple solid tumour types, including melanoma [72]. Targeted therapies are a fixed selective pressure so outgrowth of drug resistant subclones is largely inevitable. Combining targeted (or cytotoxic) drugs simply provides a different, but still fixed, selective pressure. Immunotherapy works differently. Speculating on this, successful immunotherapies may not be a fixed selective pressure, rather a “reprogramming” of the immune system i.e. “a dynamic solution to a dynamic problem”. Intermittent targeted therapy may mitigate the problem of a fixed selective pressure. In mice intermittent therapy gives an alternating selective pressure that prevents emergence of a resistant population [44].

To summarize, combining targeted drugs rationally may delay but will not prevent drug resistance in advanced solid tumours. The challenges for rationally combining targeted drugs are significant: dosing, targeting and tumour evolution. Immunotherapies may circumvent tumour evolution, perhaps because they do not provide a fixed selective pressure. There will always be a need for targeted drugs (and cytotoxics). However, the development and use of better drugs must be informed by greater understanding of disease biology.

### News in immunotherapy

New therapies for metastatic melanoma, such as cytotoxic T-lymphocyte associated antigen-4 (CTLA-4) and programmed cell death protein 1 (PD-1)/programmed cell death ligand 1 (PD-L1) pathway blocking antibodies have yielded promising results, changing the continuously evolving landscape of therapeutic options for patients with this disease. Unlike the CTLA-4 antibody blockade, which is thought to act in secondary lymphoid structures, the PD-1/PD-L1 antibodies aim to potentiate the antitumor T cell response at the tumor site, by impairing the interaction of the inhibitory receptor PD-1 on T cells with PD-L1 expressed on tumor cells. Other monoclonal antibodies under investigation in the immunotherapy of melanoma include those targeting the receptors CD40, CD137 (4-1BB), OX40, glucocorticoid-induced tumor necrosis factor receptor (GITR), KIRs, LAG-3 and transforming growth factor beta (TGFβ). Understanding the mechanism of action of these therapies can provide new rational combinations and identify biomarkers of benefit that can result in selection of patients with a high likelihood of response. The use of new immunologic agents in monotherapy and in combinations including targeted therapies will dramatically change the current management of patients with melanoma. Considering all these options the future of therapy including adjuvant therapy in melanoma appears very promising.

Goals for adjuvant therapy have historically been the improvement of Overall Survival (OS), and Relapse-free Survival (RFS). The improvement in RFS alone is entertained only if improved OS is not attainable. Consistency of adjuvant treatment impact across risk groups (IIB/IIIA & IIIB-IV) differs as this has been analyzed for previous adjuvant therapy trials. A goal of our efforts has been to identify the right patient for therapy, to optimize anti-tumor effects or to reduce the toxicity of the treatment. Understanding mechanism(s) of action of the therapy can take to new rational combinations and identify biomarkers of benefit can give more selectivity to improve therapeutic index. Two options for adjuvant therapy exist in the US for postoperative management of patients with high-risk melanoma. Agents active in adjuvant therapy are high-dose (HD) IFNα (HDI), approved by the US FDA in 1996, and pegylated (Peg)-IFNα approved in 2011. Adjuvant trials of ipilimumab administered at 10 mg/kg compared to placebo (EORTC 18071) and of ipilimumab at 10 mg/kg or 3 mg/kg compared to high-dose IFNα (ECOG led US Intergroup E1609) are pending the outcomes of current trials. For the future, trials testing anti-PD-1, anti-PD-L1 and BRAFi, as well as BRAF plus MEKi combination as well as vaccines are being evaluated.

From 1996 there have been more than 20 trials reported evaluating various formulations, dosages, routes of dosing and durations of therapy to determine the “right” regimen of adjuvant therapy and to evaluate the “right” setting of disease for adjuvant therapy. The Cochrane Analysis of all reported adjuvant trials from 1995 to 2011 (17 Trials; 10,499 patients) showed a Hazard Ratio (HR) for RFS of 0.83 (0.78-0.87) among 10,345 pts (17 studies), an HR of 0.91 (0.85-0.97) for OS on 9927 subjects (15 studies), relapse risk reduction from 50/100 to 44/100 with a number needed to treat (NNT) for benefit in one of 16 and a reduced mortality risk from 40/100 to 37/100 with commensurate NNT of 33. The Cochrane Analysis of IFN impact on RFS across studies, showed no significant heterogeneity for individual factors that significantly affect IFN impact on RFS (Subgroup analysis and meta-regression), for dosage tested, TNM stage, year of publication and treatment duration.

A fundamental assumption of adjuvant therapy is that treatment efficacy in advanced disease predicts adjuvant impact, and that the benefit in the earlier disease setting may exceed that in the setting of advanced inoperable disease. An impact upon OS may be anticipated if the benefit upon PFS HR is ~0.70 in IIIA&B. Combinations of drugs ought to build on therapeutic mechanism of action. Biomarkers will focus applications to improve risk-benefit ratio. Earlier disease will show improved gains in mortality, recurrence-free survival, and morbidity E1684, E1690, and E1694 trials showed durable and significant impact of adjuvant IFN upon RFS and OS, but mature outcomes for RFS, distant metastasis-free survival (DMFS), and OS in EORTC 18991 Trial of Peg-IFNα × 5 years for stage III melanoma did not confirm the advantage of adjuvant therapy with no impact upon OS or DMFS.

Some points are still unresolved, such as the optimal duration of treatment and the optimal dosage of IFN. It is clear that the only trials that have shown independent statistically significant improvement in both RFS and OS are the E1684 and E1694 trials, however. In regard to the optimal duration of therapy, it is also clear that one month of high-dose IFN is ineffective in improving OS, as this was tested in the US Intergroup ECOG-led E1697 trial. This large intergroup trial tested 1 month IFNα-2b vs. observation, and demonstrated that 1 month of HD-IFN is futile in IIA-IIIA patients. The Hellenic 13A/98 and Oxford Trials compared 1 month of attenuated IFN for 1 year in IIIAB with 1 month attenuated dosage HDI. The former showed no significant difference while the latter trial has recently shown improvement in the OS of patients with one year as opposed to 1 month of full HDI. The EORTC 18991 trial tested up to 5 yrs. of Peg-IFNα-2b in IIIAB and showed a relapse reduction that was marginal as currently updated [73] with a benefit in N1 but not N2 (IIIB). In this trial RFS benefit at 3.8 years resulted 18%, significant response, but at 7.6 years becomes marginal 13%, and there was no significant impact on DMFS or OS at maturity or 3.8 years. Stage III N1 disease showed significant RFS and DMFS in 2007, but improvements at 7.6 years are no longer statistically significant while patients with stage III N2 show no significant benefit at any endpoint. Mature outcomes for EORTC 18991 confirm that N1 from ulcerated melanoma is the subset showing greatest benefit with median OS Peg-IFN vs. observation with > 9 vs. 3.7 years. For this reason the EORTC 18081 trial that will compare Peg-IFN × 2 yrs. vs. observation in ulcerated primary tumors was designed. The optimal dosage of adjuvant HD-IFN has been evaluated in relation to RFS HR of 0.61-0.72 in pivotal and sequel trials consistent w/ OS HR 0.67-0.75. Benefit across stages IIB-IIIA-IIIB (RFS/OS) was restricted to IIIA (RFS) for Peg-IFN. Duration of actual therapy with HDI and Peg-IFN remain similar even if a dose response relationship remains unclear. Peg-IFNα is an alternative approved option in U.S., but more data are needed from 18081 trial.

The first generation immune checkpoint inhibitor ipilimumab has been tested in the adjuvant setting with the EORTC 18071 trial (ipilimumab 10 mg/kg vs. placebo) where RFS is the primary endpoint. Data is due in mid-2014 and the US Intergroup E1609 (ipilimumab 10 mg/kg or 3 mg/kg vs. HD-IFN (HDI) evaluates this treatment effect in relation to co-primary endpoints of OS & RFS. Closure of this trial is anticipated to also occur in mid-2014. This phase 3 trial of ipilimumab vs. HDI has been designed to verify the advantage of ipilimumab at the higher dosage of 10 mg/kg, and also to evaluate the lower US FDA approved dosage of 3 mg/kg of anti-CTLA-4 Ab as adjuvant therapy vs. HD-IFN.

New options of adjuvant therapy include a range of drug combinations with HD-IFNα. The combination of ipilimumab and HD-IFNα was demonstrated to be active and potentially additive in phase 2 trial setting for metastatic melanoma. An objective response rate of 26% with 21 months median OS and 6.4 months median PFS was obtained [74]. A phase 2 trial of ipilimumab at two different doses (3 or 10 mg/kg) combined in a 2x2 factorial design with HD-IFNα is in progress (ECOG-ACRIN trial E3611). Neoadjuvant evaluations of IFN at high dosage, ipilimumab at high dosage (10 mg/kg, UPCI 08–144) have been completed and published [75]. Currently, a neoadjuvant trial evaluating the combination of ipilimumab and IFNα is in progress (UPCI 11–063) and a trial of anti-PD-1 and IFN is designed and awaiting activation. In BRAF mutated melanoma patients the combinations of BRAFi and IFNα are under investigation through the University of Pittsburgh SPORE Program in Melanoma and Skin Cancer designed to treat BRAF-mutated patients with BRAFi followed by IFN (UPCI 12–107 trial).

Data from the UPCI 08–144 trial, neoadjuvant ipilimumab in N1b, 2b, N2c, N3 melanoma patients, evidenced an increase in tumor TILs during treatment. IL-17 levels at baseline (p = 0.02), and week 6 (p = 0.06), and change (p = 0.05) were documented to correlate with GI toxicity and the occurrence of grade 3 diarrhea. Flow cytometry analysis (N = 27) shows a rise in circulating Tregs and drop in MDSC that are associated with improved PFS (p = 0.034; HR = 0.57).

Other trials have addressed the potential role of other adjuvant therapies for high-risk resected melanoma, including chemobiotherapy. This was tested in the phase 3 S0008 trial of HD-IFNα-2b vs. cisplatin, vinblastine, DTIC, IL-2 and IFN in high risk melanoma. This showed significant differences in RFS over HDI unfortunately without any differences in OS [76].

Second generation of immune checkpoint Inhibitors, namely anti-PD-1 and anti-PD-L1, have shown single agent response rates that are unprecedented and the durability of impact favors adjuvant role even considering the favorable toxicity profile. For all these reasons there is an important potential for combination of IFN and vaccines. IFN induction of PD-L1 may sensitize for greater anti-PD-1 effects but the data regarding PD-L1 expression in RLN and in IIIA, IIIB melanoma is not available. The Intergroup S1404 Adjuvant Trial to follow intergroup E1609, has been designed to compare anti-PD-1 MK3475 MAb at 10 mg/kg every 2 weeks for 6 months followed by 10 mg/kg every 3 weeks for 2 doses, with HD-IFNα-2b, 20 MU/m^2^/day IV d. 1–5 × 4 weeks, and 10 MU/m2/day SC q.o.d., three times weekly to 48 weeks. Co-primary end points of this trial are RFS and OS; secondary end points are toxicity and immune correlatives (PD-L1 expression, tumor infiltrating lymphocytes, autoimmunity etc.). Considering all these options the future of adjuvant therapy in melanoma is more promising now than ever before in history. The use of new immunologic and targeted agents in monotherapy and in combinations, with the understanding of mechanism(s) of therapy and with the definition of prognostic and predictive biomarkers will dramatically change the adjuvant therapy.

There are two types of interferons (IFN), type I including IFN (α, β, ε, κ, ω) and type II (IFN γ). IFNα was FDA approved for application as adjuvant therapy for melanoma as discussed earlier. Type I IFNs perform their function by interaction with a specific transmembrane interferon alpha receptor, IFNAR I, which activates an intracellular pathway *via* STAT1 and 2 signaling. IFNAR1 expression is affected by activated ERK that induces the ubiquitination and degradation of IFNAR1 by β transducing repeat-containing protein 2 (βTrCP2) that is a subunit of E3 ubiquitin ligase complex. As a result, negative correlation of p-ERK with IFNAR I levels is observed in melanoma cells as IFNAR1 expression is down regulated by activated ERK. Conversely, IFNAR1 expression is up-regulated in BRAFV600E mutated melanoma cells treated with BRAF inhibitor, as demonstrated in pre-clinical models (Ferrone S, unpublished data). BRAF inhibition up-regulates IFNAR1 expression and enhances the functional properties of IFNα-2b by increasing its anti-proliferative activity and pro-apoptotic activity. Treatment of melanoma cells with BRAFi *in vitro* enhances the induction of HLA Class I antigen processing machinery (APM) components and melanoma cell sensitivity to recognition by cognate T cells. Thus the *in vivo* data confirm the *in vitro* results demonstrating the ability of the combination of BRAFi and IFNα-2b to exert anti-tumor activity and to prolong the survival of immunodeficient mice grafted with human BRAF mutated melanoma cells.

Although IFNα-2b is used as a standard treatment in melanoma side-effects may hamper reaching and maintaining the dose needed for maximal therapeutic effect and their occurrence can outweigh clinical benefit of IFNα treatment. To overcome this limitation in a clinical setting, the possibility of specific targeting of IFNα-2b to melanoma cells utilizing chondroitin sulfate proteoglycan 4 (CSPG4)-specific MAb as a carrier is being explored. CSPG4 is a membrane bound high molecular weight-melanoma-associated antigen (HMW-MAA) which is highly conserved through phylogenetic evolution. CSPG4 is expressed with high density on the membrane of malignant cells in various types of tumors including melanoma (~85%), glioma (~70%), head and neck squamous cell carcinoma (~60%), basal breast carcinoma (~80%), mesothelioma (~50%), chordoma, chondrosarcoma and osteosarcoma (~50%) and 11q23 positive acute leukemic (~20%) specimens. CSPG4 is expressed on cancer stem cells and on tumor activated pericytes, and has a restricted distribution in normal tissues. Because of its high expression on melanoma cells CSPG4 can be a useful delivery target for toxin conjugates or cytokines. Combination of IFNα delivery *via* IFNα-conjugated to CSPG4-specific MAb and up-regulation of HLA Class I antigen expression on M21 BRAF-mutated melanoma cell line treated with BRAFi suggests the feasibility of this approach for clinical application [77].

Concluding, ERK activation level correlates with IFNAR1 expression in melanoma and IFNAR1 level correlates with HLA class I expression. CSPG4-specific monoclonal antibodies can be useful carriers to deliver IFNα-2b to melanoma cells. BRAF inhibition enhances IFNAR1 expression in melanoma cells with a constitutively active mutated BRAF and enhances the functional activity of IFNα-2b suggesting that metastatic BRAF-mutated melanoma patients could benefit from the combination of IFN and BRAF inhibitor treatment.

Immune escape strategies of melanoma involve tumor cells, the tumor micro milieu and tumor infiltrating immune cells. Mechanisms linked to tumor cells can be the deficient expression and function of the MHC class I complex and components of the antigen processing machinery (APM), IFN signaling or other signaling pathway alterations. HLA class I abnormalities can be due to the total HLA class I antigen loss, HLA class I down-regulation, selective loss or down-regulation of HLA class I allospecificities and decreased level or loss of a single or multiple APM components [78]. These alterations are more frequently observed in metastases when compared to primary tumor lesions, are associated with disease progression, and also correlate with tumor staging/grading, poor prognosis and reduced patient survival. In addition, HLA class I APM deficiencies could be induced during immunotherapy including adoptive T cell transfer leading to the development of immune escape variants. Different molecular mechanisms such as structural alterations of APM genes (mutations, deletions or LOH), epigenetic control, transcriptional or post trascriptional modifications can be responsible for HLA class I defects. While structural alterations and epigenetic silencing are rare events transcriptional down-regulation is often responsible for impaired APM expression. In addition, post-transcriptional modifications, which are less frequent than the transcriptional control, could also regulate APM molecule expression. For example, down-regulation of mRNA and protein expression or proteasomal degradation of (Transporter associated with Antigen Processing) TAP1 in tumor cells leads to reduced surface expression of MHC class I antigens. Impaired TAP1 expression could also be directly associated with deficient TAP2 expression since TAP1 exerts a stabilizing function for the TAP1/TAP2 heterodimer.

A role of microRNAs (miRs) is suggested in the discordant expression of transcriptional control of APM components. Strategies used for identification of HLA/APM-specific miRs include miR array analysis of cell lines/tissues with aberrant and discordant HLA/TAP1 mRNA and protein expression, *in silico* prediction of candidate miRs, binding of miRs to 3‘-UTRs (miTRAP, enrichment of miRs, luciferase promoter analysis) and functional assays. The results lead to the identification of a number of APM-specific miRs including miR-200a*. An inverse correlation of TAP1 and miR-200a* expression was observed in human embryonic kidney HEK293T cells and melanoma cells. Overexpression of miR-200* corresponds to a reduced expression of TAP1 that correlates with reduced HLA class I surface expression which in turn enhanced NK cell recognition *via* targeting MEKK2-MEK5-ERK5 pathway (Seliger B., unpublished data). These data suggests that lower levels of HLA class I antigens in melanoma cells might be the consequence of down-regulation of TAP1 protein expression *via* TAP1-specific miRs [78].

Immune escape can be also mediated by altered signaling pathways in melanoma. Strong evidence suggests a link between IFN signaling and HLA class I APM components expression. Different mechanisms of IFN resistance such as defects in members of IFN signaling pathway have been identified [79]. In addition, modulation of basal HLA class I APM components expression by impaired IFN signaling has been implicated. Possible defects of the type I and II IFN signaling pathways involve IFN receptors, JAK/STAT signaling components, interferon regulatory factors (IRFs) or IFN-responsive elements. Lack of JAK2 expression seems to correlate with impaired IFNγ activity on tumor cells. Correlation of JAK2 function with IFNγ response of melanoma cells was demonstrated by restoration of IFN function by JAK2 overexpression in JAK2-deficient melanoma cells. Furthermore, HLA class I APM components expression was enhanced by JAK2 transfection in JAK2-negative cells. The JAK2 inhibitor-mediated down-regulation of APM and HLA class I surface expression and suppression of HLA class I APM components expression by JAK2-specific shRNA was also shown. These data demonstrate a close correlation between HLA class I APM expression and JAK signaling. Interestingly, similar results were also found by modulation of STAT1 [80].

HLA class I antigens are also influenced by tumor suppressor and oncogene (e.g., BRAF) and signal transduction activity that could cause a reduced sensitivity to T cell-mediated cytotoxicity [81]. The BRAFi vemurafenib is able to restore HLA class I APM expression and immune response suggesting a link between immune evasion and immune recognition in BRAF-mutated melanoma. B-RAF and N-RAS signaling have effect on HLA class I APM and immune response in melanoma due to altered expression of transcription factors. The transcription factor E2F1 regulates the expression of tapasin, another protein playing a role in the peptide-loading complex of MHC class I [82]. In addition, activation of the cAMP response element binding protein (CREB) by phosphorylation, which is frequently overexpressed in melanoma cells, demonstrated a CREB-mediated regulation of genes involved in different cellular processes. Thus, the underlying mechanisms of HLA class I abnormalities are diverse and a better understanding of this complexity might help to revert these deficiencies.

Immune checkpoint receptors are inhibitory molecules (CTLA-4, PD-1, Tim-3, and Lag-3) that are up-regulated on activated T cells to terminate effector T cell responses and restore immune homeostasis. Sustained expression of these molecules on T cells leads to T cell dysfunction/exhaustion, while their expression on T regulatory cells (Tregs) promotes suppressor function. Blocking interaction between the inhibitory check point receptors and their ligands become an attractive strategy for cancer immunotherapy. Tim-3 is a member of T cell immunoglobulin and mucin domain 3 (Tim-3) family of proteins which are selectively up-regulated on CD4^+^ and CD8^+^ T cells activated to differentiate to IFNγ-secreting phenotype. Tim-3 has been shown to interact with a natural ligand Galectin-9 that is up-regulated by IFNγ and expressed on many tumors. Tim-3 mediated response also depends on phosphatidylserine (PtdSer) and High-Mobility Group Box 1(HMGB1) protein that is actively secreted after cell stress and plays a role in inflammation and immune responses. In cytoplasmic tail Tim-3 has 6 tyrosine residues that can be phosphorylated and can interact with proteins involved in signal transduction. High frequency of T cells co-expressing CD8^+^Tim-3^+^ PD-1^+^ among tumor infiltrating lymphocytes has been identified and Tim-3 is a marker of the most exhausted CD8^+^ TILs. In syngeneic transplantable tumor models, anti-Tim-3 antibody synergizes with anti-PD-L1 antibody to suppress tumor growth. *In vitro* studies showed that blockade of both the Tim-3 and PD-1 pathways restores effector function of CD8^+^ T cells. Tim-3 blockade increases frequency of proliferating tumor-reactive T cells and frequency of cytokine producing tumor-reactive T cells [83]. Tim-3 is highly expressed on the transcription factor forkhead box p3 (FoxP3^+^) positive Tregs. Tim-3^+^ Tregs are present in tumor tissue and Tim-3^+^ Foxp3^+^ Tregs are potent suppressors of the local immune response. Tim-3^+^ Tregs secrete perforin and granzymes A and B as cytotoxic effector molecules to kill or deliver a negative signal to responder T cells. Tim-3/PD-L1 blockade *in vivo* down-modulates genes associated with Tregs suppressor function. Presence of Tim-3^+^ Tregs precedes the appearance of Tim-3^+^ exhausted CD8 TILs implying that Tim-3^+^ Tregs may drive T cell exhaustion process. In absence of Foxp3^+^ Tregs alterations in phenotype of CD8 TILs and the emergence of Tim-3^+^ PD-1^−^ CD8 TILs have been observed [84].

Since CD4^+^ and CD8^+^ T cell populations emerging post Tregs depletion exhibit properties of effector T cells and express Tim-3, they could be subject to Tim-3-mediated negative regulation. Indeed, Tregs depletion with a single administration of diphtheria toxin (DT) delayed colon carcinoma CT26 cells tumor growth transiently in syngeneic transplantable tumor model and administration of anti-Tim-3 antibody to Tregs depleted mice resulted in significant and sustained tumor regression. The synergistic effect of Tregs depletion and Tim-3 blockade has been repeatedly seen in MC38 colon adenocarcinoma and B16 melanoma mouse models. These data demonstrate that Tim-3 blockade after Tregs depletion relieves the Tim-3-expressing CD4^+^ and CD8^+^ T cells from Tim-3-mediated negative regulation, resulting in enhanced anti-tumor effector responses and control of tumor growth. These findings provide proof-of-principle for the combination of Tim-3 blockade with Tregs-targeted therapies for cancer treatment. IL-27 is a potent inducer of Tim-3 and in the absence of IL-27 signaling *in vivo* there is a defective Tim-3 expression and delayed tumor growth, potentially due to improved effector T cell function.

Several issues should be addressed for clinical development of Tim-3 targeted therapy. A confounding issue is the selection of an isotype of a candidate anti-Tim-3 antibody (IgG_4_ or IgG_1_). Another factor is the ability to block specific ligand interaction among a number of Tim-3 ligands that have been proposed (Galectin-9, PtdSer or HMGB1). Although the use of anti-Tim-3 MAb as a single agent seems promising, the determination which agents will work best with Tim-3 blocking MAb (e.g., anti-PD-1/PD-L1 or Lag-3) will drive development of combination therapy.

The tumor microenvironment offers multiple targets for immune system manipulation. Combined interventions targeting different mechanisms within the microenvironment are feasible and might result in more effective therapeutic efficacy. Immune-potentiating monoclonal antibodies can function as agonists of immune-stimulating receptors (CD137, CD40, OX40, GITR, CD27) or as antagonists of immune inhibitory receptors (CTLA-4, PD-1, B7-H1, BTLA, TGF-β, IL-10), and early data have suggested clinical utility. CD137 (also known as 4-1BB) is a member of the tumor necrosis factor (TNF) receptor superfamily, and it is an activation-induced T cell co-stimulator molecule. CD137 is a surface glycoprotein and it is mainly expressed on activated CD4^+^ and CD8^+^ T cells, activated B cells, and natural killer (NK) cells but can also be found on resting monocytes and dendritic cells (DCs). Its cognate ligand CD137L is expressed on APCs including B cells, monocyte/macrophages and DCs. As a co-stimulatory molecule, CD137 is involved in the activation and survival of CD4^+^, CD8^+^, and NK cells. CD137 is also involved in T cell proliferation, inhibition of apoptosis, enhances cytotoxic activity, and influences cytokine production.

Development of CD137-specific MAb began in 1997, and anti-human CD137 agonist MAbs are undergoing testing in phase 1 and 2 clinical trials. Treatment with anti-CD137 MAb can overcome tumor antigen tolerance as it affects memory CD8^+^ T cell resulting in antigen-independent activation. Treatment of activated CTLs with anti-human CD137 agonist results in resistance to apoptosis, improved proliferation, augmented effector function, and improved differentiation to memory cells. Treatment of activated NK cells with anti-human CD137 agonist MAb activates early cytokine production and early tumor cell cytotoxicity. Overall, stimulation of TILs and NK cells with anti-CD137 increased their cytolytic activity for tumor cells. NK cells upon Fc-receptor triggering, for example by antibody-bound tumor cells (e.g., trastuzumab), up-regulate the inducible co-stimulatory molecule CD137. Thus the cytotoxic function of activated NK cells can be enhanced by their exposure to an agonistic MAb against CD137 which can synergize with treatment with rituximab, trastuzumab and cetuximab to improve anti-tumor response.

Tumors are known to be hypoxic and the expression of CD137 has been demonstrated in tumors at 1% O_2_ conditions suggesting that expression on endothelial cells could be driven by hypoxia [85]. Moreover, treatment with anti-CD137 MAb resulted in enhanced expression levels of ICAM-1 and VCAM-1 on tumor endothelial cells. Considering, that hipoxia inducible factor (HIF)α is produced in response to hypoxic conditions, the HIF1α pathway becomes activated in tumors. Consequently, HIF1α is able to induce CD137 expression on antigen-primed TILs.

Systemic effects of the anti-CD137 MAb have also been demonstrated in mouse models. The CD137 protein itself lacks any known intrinsic enzymatic activity and has been shown to use TNF-receptor-associated-factor (TRAF)-1, TRAF-2 and TRAF-3 as downstream molecules to translate signals towards the cell interior [86]. TRAF2 has been shown to function as an E3 ubiquitin ligase that ubiquitinates itself and other substrates. The Lysine (K)63-Polyubiquitynation of TRAF-2 is likely to be a key step in the understanding of the physiology of CD137. *In vitro* and *in vivo* experiments documented that the CD137-dependent K63-polyubiquitination activates the transcription factors such as nuclear factor (NF)-kB and activator protein 1 (AP-1) and thereby modifies expression of many genes at a transcriptional level. The signals through CD137 ligand are also mediated by protein tyrosine kinase p38 MAPK. T cell homing into tumor lesions inducible with treatment with anti-CD137 agonist MAb was demonstrated in mouse model. Inhibition studies using anti-ICAM-1 and anti-VCAM-1 antibodies showed that the stimulation of ICAM-1 and VCAM-1 expression on tumor endothelial cells by anti-CD137 Ab was responsible for the enhanced T cell migration into tumor tissue. Anti-human CD137 MAb tested in mouse model as monotherapy resulted in systemic effects. In addition, anti-CD137 MAb effect was synergistic with other immunostimulatory monoclonal antibodies, adoptive T cell therapy, and conventional chemo- and radiotherapy. Triple combination treatment obtained important tumor regression and a significant impact on overall survival [87]. Significant impact of the triple therapy was evidenced in CD8 TILs count. Growing evidence indicates that anti-CD137 monoclonal antibodies possess strong anti-tumor properties due to their powerful capability to activate CD8^+^ T cells, to produce interferon IFNγ, and to induce cytolytic effector molecules. Currently, anti-CD137 MAb (BMS-663513) is being evaluated in several clinical trials in patients with solid tumors, including melanoma, renal carcinoma, ovarian cancer, and B-cell malignancies.

Adoptive Cell Therapy (ACT) using Tumor Infiltrating Lymphocytes (TILs) has become more successful with an observed clinical response rate from 10 to 70% in patients with metastatic melanoma and its use is spreading. TILs are re-infused after isolation from fresh tumor specimens, enrichment and expansion with IL-2 plus anti-CD3 antibody in the presence of feeder cells. Pretreatment leukocyte depletion enhances TILs therapy clinical outcomes. Radiotherapy and chemotherapy have been used for leukocyte depletion and can be either non-myeloablative or myeloablative. TILs and non-myeloablative lymphocyte depleting chemotherapy for metastatic melanoma have resulted in significant clinical responses [88]. In a recent clinical trial of TIL therapy among 57 patients treated, 5 CRs and 18 PRs were obtained; medianOS of non-responder patients was 6.1 months, while medianOS of all the patients was 15.2 months [89]. The median OS of responder patients was not reached at a median follow-up time of more than 50 months. Interestingly, shorter duration of expansion, greater expansion and infusion of more CD8^+^ cells is associated with better clinical outcomes.

Improved molecular biology techniques have increased the feasibility for the clinical application of genetically engineered T cells which have been designed to express tumor antigen specificity. Two types of engineered T cells have been developed to overcome poor antigenicity of the tumor and to generate T cells with high avidity for tumor-specific antigens, i.e., high-affinity TCRs and Chimeric Antigen Receptors (CAR). In the first approach T cells are being transduced to express natural αβTCR heterodimers of known specificity and avidity for tumor antigens. Preliminary results of ACT with genetically engineered T cells have been promising. Treatment with genetically engineered lymphocytes expressing NY-ESO-1 antigen specific TCRs resulted in clinical responses in 5 of 11 melanoma and in 4 of 6 synovial sarcoma patients [90]. Genetically engineered lymphocytes reactive with MAGE-A3 obtained clinical responses in 5 of 9 treated patients [91].

CAR therapy involves the generation of T cells with chimeric receptors that have antibody-based external receptor structures and cytosolic domains that encode signal transduction modules of the T cell receptor. These constructs can function to retarget T cells *in vitro* in an MHC-unrestricted manner to attack the tumor while retaining MHC-restricted specificity for the endogenous TCR. Clinical results of CAR T cell therapy have been encouraging. Among 8 patients with B-lineage hematologic malignances treated at the NCI with autologous anti-CD19 CAR T cells, 6 clinical responses were achieved [92]. Other CAR T cells being tested in clinical trials are anti-CD20 in B cell lymphoma, anti-GD2 in neuroblastoma and osteosarcoma, and anti-ERBB2 (HER-2/neu) in colon cancer. CAR T cells in development include anti-CD22, anti-CD23, anti-CD70, anti-Immunoglobulin Kappa light chain, anti-B cell maturation antigen (BCMA) for multiple myeloma, anti-Glypican 3 for hepatocellular carcinoma, and anti-Erythopoietin-producing hepatocellular carcinoma A (EphA2) for glioblastoma.

It may also be possible to improve adoptive cellular therapy by making use of T cells with less differentiated phenotype. The population of memory T cells with enhanced stem cell-like (TSCM) qualities compared to known memory populations displays enhanced self-renewal and multipotent capacity to derive central memory effector memory and effector T cells. In mouse models adoptively transferred Mesothelin-specific TSCM cells demonstrated increased proliferative capacity, more efficiently reconstituted immunodeficient hosts and mediated superior anti-tumor responses in a humanized mouse model [93].

Since genetically engineered T cells are clinically effective and are relatively easy to manufacture and some of the technologies related to T cell engineering are protected by intellectual property, companies have become interested in developing commercial T cell therapies. As a result of this increased interest on ACT manufacturing methods for generating cellular anti-cancer therapies are improving.

Ipilimumab, the anti-CTLA-4 MAb, induces tumor necrosis, infiltrates of lymphocytes and vasculopathy. Angiogenesis has an important role in immune suppression, and in a study of melanoma patients treated with ipilimumab changes in distribution of VEGF at baseline and week 12 were observed. Changes of VEGF levels were observed in some melanoma patients relative to treatment, but these were not correlated with clinical outcomes. Baseline VEGF value correlated with overall survival. Considering a VEGF cut-off of 43 pg/ml, an association of baseline VEGF with clinical response at week 24 for 157 patients treated ipilimumab was evidenced (p = 0.019). As a result, ipilimumab was tested in association with bevacizumab in a phase 1 clinical trial. Patients were divided in different cohorts: cohort 1: 10 mg/kg ipilimumab plus 7.5 mg/kg bevacizumab, cohort 2: 10 mg/kg ipilimumab plus 15 mg/kg bevacizumab (dose expansion), cohort 3: 3 mg/kg ipilimumab plus 7.5 mg/kg bevacizumab, cohort 4: 3 mg/kg ipilimumab plus 15 mg/kg bevacizumab with the induction phase every 3 weeks × 4 cycles and the maintenance phase of bevacizumab continued every 3 weeks, and ipilimumab continued every 3 months. Grade >3 toxicities included hypertension, giant cell (temporal) arteritis, hepatitis and bilateral uveitis/retinitis. Best objective response rate (BORR) (Complete Response and Partial responses) resulted in 17.4% and durable stable disease (≥6 months) were observed in 50% of the patients. At maximum tolerated dose (MTD) Complete Response/Partial responses were 35.3% and durable Stable Disease (≥6 months) resulted in 53% of patients (analysis of 17 patients). Median follow up time was 17.3 months. Median PFS was 9.0 months, 95% CI (5.5 to 14.5 months). In the subgroup analysis, according with the different cohorts, PFS resulted in C1: 6.4 months, in C2: 14.5 months, in C3: 6.6 months and in C4: 7.6 months. Median overall survival resulted 25.1 months, 95% CI (12.7 to ∞). Median OS for the treatment population including MTD was 25.1 months. There was apparent increase in central memory phenotypes for both CD4 and CD8 cells with the addition of bevacizumab not seen to such an extent with ipilimumab alone.

Morphologic changes in tumor endothelium and blood vessels in post-treatment biopsies were also observed as a function of treatment. Endothelia activation was associated with intense immune cell infiltrates. In pilot studies to identify potential targets of the immune responses, Galectin-1 and Galectin-3 were identified. Galectin-1, −3 and −9 specific antibodies were commonly detected in melanoma patients treated with ipilimumab and bevacizumab and frequently increased as function of treatment. Targeting VEGF can have potential synergies with CTLA-4 blockade inducing immune mediated vasculopathy, immune modulatory effects of VEGF, decreased DCs maturation and T cell trafficking. The combination of ipilimumab and bevacizumab may be an effective therapy in metastatic melanoma, but it needs further investigations in randomized phase 2 clinical trials [94].

### Tumor microenvironment and biomarkers

Markers that accurately predict responding patients would be of the greatest clinical utility in all types of therapy. Once a cancer biomarker has been validated, it can be used to diagnose, to define disease risk or to tailor treatments for an individual patient. The identification of biomarkers for targeted therapy will help clinicians to stratify patients into molecular subgroups, which facilitate the selection of therapy. Several studies suggest that immune-based correlates to an anti-tumor response in clinical trials of cancer immunotherapy could also be found. The assays that could define the potency of the immunomodulatory agent or biomarkers that may directly assess immune destruction at the targeted tumor, thus directly reflecting the anti-tumor response are of great need. Many candidate biomarkers are worthy of exploration but they require appropriately powered specimen cohorts and clinical trials to determine their clinical utility before they are used in clinic.

Understanding how tumor rejection occurs will help to optimize existing immunotherapies and develop new effective anti-cancer therapeutic approaches. Adoptive cell transfer therapy (ACT) has been a successful approach to treat melanoma patients. In order to identify factors in tissue infiltrating lymphocytes (TILs), tumor or the host that associate with clinical response, TILs from 142 patients enrolled in five adoptive cell therapy trials, 113 melanoma metastases and 15 melanoma cell lines derived from the 15 melanoma metastases were analyzed. Gene expression profiling of TILs selected from patients that achieved a complete response (CR), a partial response (PR) or lacking response (NR) after treatment with autologous TILs and IL-2 demonstrated significant differences in expressed genes. Specifically, comparison of TILs of patients with CR to TILs of NR patients identified 61 highly differentially expressed genes including apoptosis-related genes, chemokine receptors, cytokines and others.

A panel of 113 melanoma metastases from patients with CR, PR or NR has also been compared by gene expression and 15 cell lines derived from those metastases using array Comparative Genomic Hybridization (aCGH) were characterized. Negative correlation between the mRNA level for nitric oxide synthase 1 (NOS1) and patients response to ACT therapy was observed. High NOS1 expression also correlated with gene amplification of NOS1 locus in the segment 12q22-24. NOS1 functional impact on immune response was further validated *in vitro* in melanoma cell lines co-cultured with normal donor PBMC stimulated with IFNα using STAT1 phosphorylation assay. High NOS1 producing melanoma cell lines demonstrated significant inhibition of pSTAT1 in PBMC suggesting that NOS1 expression links immune dysfunction in circulating immune cells with an identifiable genotype of melanoma. The direct impact of NOS1 was further tested by i*n vitro* assays using a mimic of NOS1 products Nitric Oxide (NO) which directly inhibits IFNα signaling [95]. This data suggest a critical role for NOS1 for melanoma induced immune suppression and that melanoma cell-derived NO is a crucial modulator of immune function in the tumor microenvironment and provides a potentially novel target for immunotherapy. NOS1 status could also serve as a potentially predictive biomarker of immune responsiveness to adoptive cell therapy.

In addition, to distinct gene expression signature of TILs associated with complete tumor regression host genetic factors which might determine predisposition to tumor response were explored. Interferon Regulatory Factor (IRF)-5 is a key transcription factor in IFN type I pathway and is a critical mediator of host immunity. IRF-5 signaling plays important role in development of systemic lupus erythromatosus (SLE) autoimmunity by inducing apoptosis, and up-regulating inflammatory cytokines (IL-6, IL-12, TNFα, etc.) and IFNα/β secretion. Polymorphisms in IRF5 resulting in enhanced IRF-5 function have been associated with SLE [96]. Similarly, polymorphic SNP variants may contribute to the diverse clinical outcome for cancer patients treated with immunotherapy. Genetic polymorphism analysis of IRF-5 in patients undergoing ACT therapy demonstrated significant association with clinical outcome. Differences in genetic variants of IRF-5 in responders vs. non-responders suggest that IRF-5 genotype may influence immune responsiveness by affecting the intrinsic biology of melanoma [97].

Extended polymorphism analysis using Genome Wide Association Study (GWAS) comparing CR vs. NR patients demonstrated G-protein coupled receptor kinase 5 (GRK5) polymorphisms to have one of the highest association with outcome. Several studies have shown a broad role for GRK5 in cell signaling and it has been proposed to be a critical kinase in the pathogenesis of several diseases including cancer. GRK5 has broad role in cell signaling including its function as a mediator in inflammation. GRK5 can be a positive regulator of LPS-induced inflammatory cytokine and chemokine production *in vivo via* IkBα-NFκB signaling pathway. Both IκBα phosphorylation and gene expression were significantly inhibited in the GRK5 knock out (KO) mice compared to the wild type (WT mice). In addition, inflammatory response, and thymic apoptosis were markedly reduced in GRK5 deficient mice. GRK5 and another kinase in the GRK family GRK6 regulate Wnt/β-catenin signaling by phosphorylating low density lipoprotein receptor-related protein 6 (LRP6). Phosphorylation of TP53/p53 by GRK5 inhibits TP53/p53-mediated apoptosis and phosphorylation of adaptor protein ST13 controls internalization of the chemokine receptors (CCR3, CXCR2, 4 and 6). Together, the data implicate GRK5 as an important molecular target in melanoma.

Environmental factors such as gut commensal bacteria and their immune regulatory function in anti-cancer immune response were explored in animal model. Experimental evidence in models of autoimmunity and infection indicated that systemic innate and adaptive immune responses are influenced by gut commensal bacteria and they are impaired by treatment with antibiotics. In mouse model, SC transplanted tumor responded to anti-IL-10R antibody administration following intratumoral CpG treatment resulting in tumor rejection. However, pre-treatment with antibiotic (vancomycin, imipenem, and neomycin) to create bacterial free gut abrogated efficacy of anti-IL-10R antibody and CpG treatment [98]. This data provide evidence that intestinal bacteria can influence the inflammatory and anti-tumor response.

In summary, tumor rejection remains a multifactorial phenomenon and involves the genetic, epigenetic, phenotypic characteristics of cancer cells, immune cells, host factors as well as an environment. Understanding these interactions might have implications for classification of patient for specific therapies.

Tumor cells use different mechanisms to escape immune recognition including antigen/MHC loss, expression of ligands for inhibitory T cell receptors on the tumor surface (e.g., PD-L1), and secretion of immunosuppressive cytokines among others [99,100]. The assessment of the immune/inflammatory infiltrates in the tumor bed could help to predict the responsiveness to immune modulating therapies, such as adoptive-cell-transfer therapy (ACT) with tumor-infiltrating lymphocytes (TILs). TILs are generated from the *in vitro* culture of tumor cells digests derived from the tumor followed by *in vitro* expansion. In most clinical trials, the measure of anti-tumor activity of the expanded TILs before infusion into the patient is secretion of IFNγ. Pretreatment lymphodepletion based on either a round of chemotherapy or on whole-body radiation is used to increase the success of ACT therapy.

An experimental approach to generate TILs involves two phases. The first phase encompasses the initial isolation of TILs from fresh tumor specimens and expansion with high dose of IL-2. Specifically, tumors were minced into fragments, digested with triple enzymes mixture of collagenase, hyaluronidase and DNase, and resulting cell suspension was washed and cells were counted. Cultures of 10^6^ cells (Tumor plus T cells)/2 ml/well in a 24 well plates were established (RPMI-1640, Human AB serum plus 6000 IU/ml IL-2) and cells were cultured for 3 – 8 weeks. In the second phase cell cultures are scaled up to generate the final infusion product. After the initial outgrowth T cells were co-cultured alone (negative control), with immobilized anti-CD3 (positive control) or with feeder cells that were either autologous tumor cells or unrelated melanoma tumor cell lines (specificity control). Quantitative IFNγ release assay was used to determine TILs effector function. The results of culturing a cohort of 36 TIL cultures were as follows [Bifulco et al. in progress]. Approximately half of TIL cultures were successfully grown and demonstrated tumor–specificity. A quarter of TIL cultures were established but they did not secrete IFNγ in response to specific tumor stimulation. Another quarter of cell cultures failed to grow.

Despite the success of ACT therapy in achieving a clinical response rate the degree of successful culture of TILs and the response among patients can greatly vary among patients. Pilot studies demonstrated promising results with this treatment approach but there are both scientific and logistic challenges to overcome to expanding the use of this therapy in clinic. Not all metastatic melanoma patients who have their tumor surgically resected are eligible for TIL therapy, as the TILs from their tumors cannot be successfully grown and expanded to sufficient numbers or lack tumor-specific function. Some patients may have T cells that have lost potency or the ability to proliferate and function poorly. Predictive biomarkers, which could be used to predict whether a tumor from a given patient will yield successfully growing TIL culture for therapy, are needed. For example, the total number and TILs composition, including the frequency of CD8 vs. CD4 T cells could have an impact on *in vitro* growth and the clinical response of ACT. The expression of a number of markers including CD27, CD28, CCR7, CD62L, CD45RA/RO, CD25 and 4-1BB/CD137 by T cells has been indicated to correlate with T-cell differentiation and activation and response. Chemokine signature, immune effector function markers and immunosuppressive factors might be important to better define the phenotypic state and competency of T cells for TILs therapy.

Immunohistochemistry (IHC) is a useful method that allows for a detailed evaluation of the tumor *in situ* and its microenvironment, including the composition of immune cell infiltrate and the pattern of distribution of infiltrating TILs (intratumoral vs. peritumoral infiltration). If properly validated, IHC analysis could enable identification of potential biomarkers that correlate with clinical outcome of ACT treated patients. Histological analysis of the formalin fixed and paraffin embedded (FFPE) tumor blocks from synchronous and metachronous melanoma biopsy was carried to evaluate the inflammatory/immune microenvironment using IHC and digital quantification of images.

The data suggest that 50% of failures to grow tumor-specific TILs are associated with the presence of a MDSC rich microenvironment (Low CD8, High CD163, and High MPO). The presence of increased numbers of Tregs and MDSCs that are major components of the immune suppressive microenvironment could promote T cell dysfunction. These cells might suppress T cell activation by multiple mechanisms, including uptake of essential amino acids for T cell activation like arginine and/or cysteine. Preliminary data suggests that the ratio between T effector cells and suppressor cells in the tumor may be indicative of the successful/unsuccessful TILs culture. However, no clear criteria and the cutoff value for the density of suppressor cells were identified. Lack of correlation between cellular components and the growth success rates suggests other factors that might predict the success rate in growing TILs. Secretion of immunosuppressive molecules like TGF*β* has also been implicated in suppressive function of myeloid cells. Other immunosuppressive factors such as the enzyme indolamine deoxygenase (IDO) and PD-L1 expression might contribute to a negative feedback mechanism in the tumor microenvironment.

Further studies are needed to characterize multiple metachronous metastases from patients for who TIL cultures cannot be established and compare with TIL cultures that have been successfully grown. These data might help to understand the mechanisms involved in the microenvironment that are conducive to the *in vitro* growth of tumor-specific TILs. The set of markers to identify such factors including a myeloid-enriched/T effector-poor microenvironment might help to identify patients with increased likelihood to establish tumor-specific TILs for ACT therapy. This information might also be used to develop strategies to pre-treat patients in order to augment conditions that improve recovery of tumor-specific T cells.

Over the last few years, several newly developed immune-based cancer therapies including anti-CTLA-4, anti-PD-1/PD-L1 as well as IFNα have been shown to induce clinical responses in significant numbers of patients. As a result, there is a need to identify immune biomarkers capable of predicting clinical response.

IL-2 and sIL-2R (sCD25) seem to have a role in anti-CTLA-4 monoclonal antibody efficacy. IL-2/IL-2Rβ- dependent efficacy of ipilimumab has been demonstrated in mice model. Blocking IL-2Rβ, ipilimumab efficacy improves in mice implying that sCD25 can be considered a decoy receptor with immunosuppressive functions. Furthermore, data from the analysis of 272 patients with metastatic melanoma from 9 cohorts of patients showed that sCD25high combined with LDH^high^ levels predict resistance to ipilimumab [Zitvogel et al., submitted].

A phase 1 trial studied clinical activity, safety and biomarkers of PD-L1 blockade with an engineered anti-PD-L1 antibody (MPDL3280A) in non-small cell lung cancer (NSCLC). Additional analyses from a clinical studies showed that PD-L1 is broadly expressed in NSCLC, with a prevalence of about 45% in adenocarcinomas and 50% in squamous cell carcinomas. In the ongoing phase 1a trial with MPDL3280A MAb that was administered IV every 3 weeks for up to 16 cycles in patients affected by metastatic solid tumors; patients were stratified according with PD-L1 status. The treatment resulted in ORR in 23% patients with 17% SD longer than 24 weeks and 24 weeks PFS in 45% of NSCLC patients.

Correlation between best response and PD-L1 expression in tumor tissue using IHC was observed. In tumors with PD-L1 level at IHC score 3 (≥10% tumor immune cells positive for PD-L1) ORR resulted in 83% of patients (5/6), while in patients with an IHC score of 2 and 3 (≥5% tumor immune cells positive for PD-L1) ORR was 46% (6/13). ORR was 31% in patients with a PD-L1 IHC score 1, 2 (≥1% tumor immune cells positive for PD-L1). These data show that PD-L1 expression as measured by immunohistochemistry suggested a correlation between pretreatment tumor PD-L1 expression and clinical response to anti-PD-L1 monotherapy [101]. Pre-treatment levels of PD-L1 expression were also associated with increased likelihood of response to nivolumab monotherapy in advanced melanoma, although some responses were also observed independent of PD-L1status including negative patients. However, PD-L1 expression did not correlate with response for the concurrent regimen of nivolumab and ipilimumab.

Markers predicting response to anti-CTLA-4 are not well defined but absolute lymphocyte count (ALC), immune-related adverse events (irAE), NY-ESO1 expression, and sCD25 have been implicated in several studies. A rise of the ALC during-treatment was associated with favorable response to ipilimumab monotherapy but no correlation between responses to nivolumab monotherapy or ipilimumab and nivolumab combination therapy and response and ALC status was seen.

Ulceration can be considered a biomarker of IFN sensitivity. Ulcerated melanoma can be considered as a tumor with distinct biology because it correlates with much lower survival for the tumors adjusted to Breslow thickness. Data from 2644 patients enrolled in the IFN adjuvant trials EORTC18952 and EOTRC18991 evidenced an HR of 0.75 for RFS, an HR of 0.59 for DMFS and an HR of 0.58 for OS in patients with ulcerated melanoma. Long term follow-up confirmed the survival advantage of IFN therapy in ulcerated melanoma (EORTC 18991). The meta-analysis of individual patients’ data from randomized trials, performed in 2007, confirmed that the only subgroup with a benefit from IFN therapy was observed in ulcerated melanoma. In 2014 at ASCO this was confirmed in the Individual Patient Data (IPD)-Meta-analysis of all 15 trials comparing IFN to observation, in >7500 patients [102].

Thus, IFN can be considered a “targeted therapy” for ulcerated melanoma patients because only ulcerated melanomas are IFN sensitive (OS HR 0.77), while non-ulcerated tumors are IFN insensitive (OS HR 0.98). Furthermore, the stage is important because in stage IIB- III- N1 (Sentinel Node +) patients HR for OS resulted in 0.60 while stage III-N2 (Macroscopic/Palpable Node +) HR for OS was 0.85. Preliminary results of a molecular signature showed that 6 miRNAs emerge in ulcerated patients with IFN therapy that did not relapse. Identification of predictive markers of response would be valuable to guide effective use of immunotherapies.

Expression of a subset of chemokine genes is associated with presence of CD8^+^ T cells in melanoma metastases, and a transcriptional profile encompassing expression of these genes appears to identify patients with clinical benefit from immunotherapies. Two immunologic subtypes of melanoma based on tumor microenvironment have been described: T cell rich/inflamed, expressing chemokines, CD8^+^ T cells, and a type I IFN signature; and T cell poor/non-inflamed, with lack of chemokines for T cell recruitment and low indicators of inflammation [103]. These two immunologic subtypes have different mechanisms of resistance. T cell rich/inflamed uses immune inhibitory pathways, while T cell poor/non-inflamed uses immune exclusion as a major mechanism of resistance [104].

Immune inhibitory mechanisms dominating in CD8^+^ T cell-infiltrated melanomas are PD-L1 (engages PD-1 on activated T cells), IDO (indoleamine-2, 3-dioxygenase; degrades tryptophan), CD4^+^CD25^+^FoxP3^+^Tregs (extrinsic suppression) and T cell anergy (T cell intrinsic dysfunction). In fact, the presence of Tregs and expression of PD-L1 and IDO are associated with a CD8^+^ T cell infiltrate and these factors are part of an immune-intrinsic negative feedback loop [105].

Another mechanism of resistance is T cell-intrinsic dysfunction (anergy). CD8^+^ TILs that co-express LAG3, PD-1, and 4-1BB or cytotoxic and regulatory T cell molecule (CRTAM) are defective at IL-2 production. They also show defective proliferation and blocked RAS pathway activation. However, these dysfunctional T cells still make IFNγ and Tregs-recruiting chemokines (CCL1, CCL22), arguing that they may themselves have immune regulatory properties. Most of the tumor Ag-specific T cells fall into this subset. Interventions aimed at uncoupling negative regulatory mechanisms in T cell-infiltrated melanomas are under development and are generating intense interest. These include inhibitory receptor blockade with MAbs against PD-1/PD-L1 or CTLA-4; anergy reversal with combinations of anti-41BB, anti-LAG3, and/or anti-Tim-3; IDO inhibition using small molecule inhibitors, and anti-CD25 for Tregs depletion. Anti-PD-1 MAb in metastatic melanoma generated a 30% RR in 90 patients treated [106]. In mouse models, combinatorial targeting of CTLA4 ± PD-L1 ± IDO results in improved tumor control. Synergistic permutations markedly increase the number of proliferating IL-2-producing CD8^+^ T cells in tumor microenvironment [107]. Combinatorial treatment with anti-CTLA-4 and anti-PD-1 MAbs also can have profound clinical activity in metastatic melanoma. Other combinations ongoing or planned are anti-CTLA-4 plus IDOi phase 1/2, anti-KIR plus anti-PD-1 phase 1, anti-LAG-3 plus anti-PD-1 phase 1, Vaccine plus anti-CD25, or anti-PD-1 plus IDOi.

The mechanism by which the spontaneous T cell response develops against melanoma is being elucidated. Innate immune sensing of tumors drives host type I IFN production and cross-priming of CD8^+^ T cells *via* CD8α+/−DCs [108]. Recent evidence suggests that the innate immune sensing pathway that mediates host type I IFN production is *via* the stimulator of interferon genes (STING) pathway [Woo et al., manuscript submitted]. STING agonists are being developed to induce de novo inflammation in non-T cell-infiltrated cancers. DMXAA is a mouse STING agonist, and intratumoral DMXAA promoted rejection of B16 melanoma in a mouse model. DMXAA triggered a potent CD8^+^ T cell response against tumor-expressed SIY antigen and mice that rejected tumors with DMXAA were protected against a second tumor rechallenge [Corrales et al., manuscript submitted].

Molecular mechanisms that explain why a subset of patients develops a spontaneous T cell infiltrate while another major subset does not are being pursued *via* three major hypotheses: 1) Germline genetic differences at the level of the host (polymorphisms in immune regulatory genes, e.g. in type I IFN and STING pathways); 2) Somatic differences at the level of tumor cells (distinct oncogene pathways activated in different patients or mutational landscape and antigenic repertoire); and 3) Environmental differences (intestinal microbiome /immunologic/pathogen exposure history of patients). Logical combination therapies are being pursued clinically that are highly anticipated to show improved efficacy.

In the treatment of metastatic melanoma, mutant BRAFV600E can be targeted with selective BRAF inhibitors (BRAFi) such as vemurafenib and dabrafenib. Although initial response rates are high, tumors eventually become resistant and progress [109]. Anti-CTLA-4 and anti-PD-1 antibodies, however, can induce durable responses in a small subgroup of melanoma patients [110,111]. Combining selective BRAFi and immunotherapies could prove beneficial in avoiding the short comings of the individual agents [112]. To persuade combinations of immunotherapies and selective BRAFi, the immunological consequences of selective BRAFi treatment need to be explored. Selective BRAFi might support anti-tumor immunity directly or indirectly by disarming immunosuppressive factors such as MDSC. MDSC are a heterogeneous population of myeloid cells that able to suppress innate and adaptive immunity by various mechanisms. Accumulation of MDSC has been described in patients with different malignant tumors including melanoma [113]. In peripheral blood mononuclear cells (PBMC) from patients with advanced melanoma, two phenotypically distinct MDSC subsets were recently described: monocytic (mo)MDSC which are CD45^+^Lin^−^HLA-DR^-/low^CD14^+^CD15^dim^ CD66b^−^CD33^+^CD11b^+^Arginase1^−^CD16^-/low^ and granulocytic (gr)MDSC which are CD45^+^Lin^−^HLA-DR^−^CD14^−^CD15^+^CD66b^+^ CD33^+^CD11b^+^Arginase1^+^CD16^-/low^ [114]. A significant increase in the frequency of both subsets was found in patients witch advanced melanoma as compared to healthy donors and patients with localized disease [114]. In patients responding to vemurafenib, tumor-related accumulation of both subsets was reversed. *In vitro*, vemurafenib decreased the ability of melanoma cell lines to induce moMDSC*.* While these findings indicate a beneficial role of vemurafenib in anti-tumor immunity, the effector phase of the anti-tumor response needs to be considered, too. *In vitro,* analogues of vemurafenib do not inhibit human lymphocyte function [115]. Recognition and cytotoxicity against melanoma cell lines by T cells specific for melanoma differentiation antigens (MDA) was increased by selective BRAFi treatment, that up-regulated MDA expression [115].

Analysis of tumor biopsies obtained during treatment with dabrafenib or vemurafenib also showed an enhanced MDA expression [116] and an increased infiltration of melanoma metastases by human CD4^+^ and CD8^+^ T cells, the latter associated with the reduction of tumor mass. Composition and functionality of patients’ lymphocytes remained unaffected with dabrafenib treatment [117]. Numbers of circulating T, B and NK cells did not change and *ex vivo* memory responses by CD4^+^ and CD8^+^ T cells to recall antigens were not impaired by treatment with dabrafenib. In summary, lymphocyte function seems to be unaffected by selective BRAFi while antigenicity of melanoma cells is increased. Recently, the first comprehensive, immunological study of vemurafenib and dabrafenib has been performed [118].

Surprisingly, vemurafenib but not dabrafenib decreases the number of peripheral lymphocytes in melanoma patients. Loss of lymphocytes was found to be associated with treatment rather than disease progression and did not predict survival in patients treated with selective BRAFi. Loss of peripheral lymphocytes was also independent from pre-treatment LDH value. Further analyses of lymphocyte subpopulations showed that CD4^+^ T cells, but not CD8^+^ T cells or B cells were decreased significantly during vemurafenib treatment while an increase of circulating NK cells can be observed. Phenotypically, a shift toward a naïve phenotype was observed within CD4^+^ T cells compared to pre-treatment samples. Also, CD4^+^ T cells isolated from samples obtained during vemurafenib treatment showed a decreased ability to secrete INFγ and IL-9. Thus, vemurafenib causes a selective loss of CD4^+^ T cells and changes the composition and impairs the function of peripheral CD4^+^ T cells. Vemurafenib increases the frequency of naive but decreases the frequency of central memory CD4^+^ T cells and decreases the ability of CD4^+^ T cells to secrete IL-9 and IFNγ when analyzed *ex vivo* [118]. These findings indicate a different impact of vemufafenib and dabrafenib on the human immune system which is supported by recently presented clinical data. While a phase 1 trial employing vemurafenib in combination with ipilimumab had to be discontinued due to liver toxicity, such toxicity was not observed when dabrafenib was given concomitant to ipilimumab [119]. Taken together, these clinical and translational findings indicate that different selective BRAFi can show a distinct impact on the human immune systems which needs to be considered, in particular when planning combinations of selective BRAFi and immunotherapies.

For the physician treating patients with completely resected stage II/III melanoma, a dilemma is how to advise the patient and whether to prescribe interferon or an experimental treatment. The key question is whether micrometastases, formed before the tumor was surgically removed, are present and able to grow and expand. This can be influenced by tumor intrinsic factors (oncogenes, cell type of origin or location of primary tumor) and host factors (tumor micro-environment and the immune system). Immune surveillance controls dormant tumors and, in melanoma, CD3 count and TILs correlate with improved survival in stage IIIA-IVA disease [120]. Further, the immunoscore and immune profiling is prognostic in multiple tumor types [121]*.* However, RNA profiling has been challenging in primary melanoma tumors because clinical standards dictate that the entire tumor be FFPE, making it difficult to isolate high quality RNA [122].

NanoString is a probe-based technology specifically designed to quantify mRNA transcripts in partially degraded RNA [123]. Using NanoString, we quantified mRNA copy number for 446 genes selected using a hypothesis driven approach based on literature review in a training set of primary tumors (n = 40) [124]. NanoString results were confirmed by immunohistochemistry. Based on this data, using linear regression models, we defined a 53-immune gene panel correlating with recurrence free and disease specific survival in the training set. This gene panel was then validated in a second independent population (n = 48). These 53-genes have high overlap with a co-expression network identified using unbiased methods using expression data available in GEO, Gene Expression Omnibus (GEO), a public functional genomics data repository supporting MIAME-compliant data submissions. Key nodes in a Baysian network defined using the GEO expression data identify Th1 processes, TCR and BCR activation, and CD2 as critical modulatory pathways. Protein levels of CD2 measured by IHC were also found to correlate with non-progression and prolonged survival. Analysis of immune gene expression in primary melanoma tumors is likely to yield valuable biomarkers and the proposed 53-gene panel should be studied prospectively in larger studies.
